# Application of *Brassica juncea* and *Raphanus sativus* Sprout Extracts as Active Agents in Chitosan-Based Edible Coatings: Evaluation of Physicochemical and Biological Properties

**DOI:** 10.3390/polym18020252

**Published:** 2026-01-16

**Authors:** Arash Moeini, Roméo Arago Dougué Kentsop, Aspen Beals, Monica Mattana, Massimiliano Marvasi, Lucie Coquard, Marianna Gregorio, Judyta Cielecka-Piontek, Annamaria Genga, Aleksandra Nesic, Giovanna Lo Vecchio, Sarai Agustin Salazar, Thomas Becker, Pierfrancesco Cerruti

**Affiliations:** 1 Chair of Brewing and Beverage Technology, TUM School of Life Sciences, Technical University of Munich, Weihenstephaner Steig 20, 85354 Freising, Germany; 2 Institute of Agricultural Biology and Biotechnology, National Research Council of Italy (CNR-IBBA), Via Alfonso Corti 12, 20133 Milan, Italy; 3 Department of Biology, University of Florence, Via Madonna del Piano 6, Sesto Fiorentino, 50019 Florence, Italy; 4 Clermont Auvergne INP, Université Clermont Auvergne, 27 Rue Roche Genès, 63170 Aubière, France; 5 Department of Pharmacognosy and Biomaterials, Poznan University of Medical Sciences, Rokietnicka 3, 60-806 Poznan, Poland; 6 Vinca Institute of Nuclear Sciences, University of Belgrade, Mike Petrovica Alasa 12-14, 11351 Belgrade, Serbia; 7 Institute of Polymers, Composites and Biomaterials (CNR-IPCB), Via Campi Flegrei 34, 80078 Pozzuoli, Italy

**Keywords:** biopolymers, encapsulation, antioxidant activity, sprout extracts, antimicrobial activity

## Abstract

The use of natural bioactive compounds in edible coatings provides a sustainable approach to reducing food spoilage and meeting consumer demand for safer food preservation. In this study, bioactive extracts from *Brassica juncea* (green mustard, GM) and Raphanus sativus (radish tango, RT) sprouts were encapsulated into zein/chitosan (Z/CH) microparticles (MPs) using a complex coacervation–based encapsulation approach. The encapsulated microparticles (MPs), characterized by FTIR and UV-Vis spectroscopy, demonstrated a high loading efficiency of up to 90% and maintained their antioxidant activity for up to 168 h. TGA and SEM tests confirmed that the edible films produced by incorporating these microparticles (MPs) into polyvinyl alcohol (PVA) and chitosan (CH) matrices had a more uniform microstructure and enhanced heat stability. The Z/CH/RT6:PVA (1:2) and Z/CH/GM6:CH (1:1) formulations of the films showed significant antioxidant and antibacterial action, with up to 22.4% DPPH inhibition and a 1-log decrease in *Salmonella enterica* CFU, respectively. Overall, the results underscore the promise of sprout-derived microparticles as components for developing active, biodegradable packaging films with improved functional properties.

## 1. Introduction

Packaging protects food from mechanical stress and limits chemical or microbial deterioration, preserving quality and extending shelf life during storage and distribution [1]. It also facilitates handling, transport, and consumer convenience. However, the dominance of petroleum-based plastics in the packaging sector has generated severe environmental and economic concerns. Non-biodegradable plastics persist in air, water, and soil for decades [2] and only about one-quarter of packaging plastics are recycled, while most remain non-degradable [3]. In line with the EU 2030 sustainability agenda, replacing fossil-based plastics with bio-derived and biodegradable alternatives is therefore a global priority [4]. Edible coatings, thin, invisible biopolymer layers applied directly to food surfaces, provide an environmentally friendly means to control moisture migration, oxidation, and microbial growth. By incorporating safe plasticizers, antioxidants, or antimicrobials, they can also enhance texture, appearance, and shelf life [5]. Such coatings must comply with food safety regulations and typically employ renewable biopolymers, polysaccharides, proteins, or lipids, combined with food-grade additives, owing to their biodegradability and non-toxicity [6]. Among polysaccharides, chitosan (CH), obtained by deacetylating chitin from crustaceans and insects, forms cohesive films and exhibits inherent antimicrobial activity against food-borne microorganisms [7]. Recognized as safe by the U.S. FDA, chitosan coatings can delay ripening and maintain firmness in fruits through their strong surface adhesion and antibacterial properties [8]. Polyvinyl alcohol (PVA), a water-soluble, non-toxic, and biodegradable polymer approved by both the FDA and EMA, offers excellent mechanical strength and compatibility with hydrophilic biopolymers [9]. When blended with chitosan, PVA enhances flexibility, moisture retention, and film-forming behavior [10]. Zein (Z), an alcohol-soluble protein derived from maize endosperm and a byproduct of bioethanol production, is rich in hydrophobic amino acids, enabling the formation of strong barrier films against water vapor and oils [11,12]. The integration of bioactive compounds into such polymeric matrices has broadened the scope of biopolymers from passive to active packaging, where incorporated natural agents provide antioxidant or antimicrobial protection [13,14,15]. Plant-derived extracts are particularly attractive for this purpose because they combine high bioactivity with consumer acceptance [16,17,18]. Their inclusion in edible coatings improves food quality and safety through synergistic antioxidant and antimicrobial effects. For instance, rosemary essential oil encapsulated in fenugreek-mucilage films prolonged apple shelf life by approximately 30 days [19]. Sprouts from green mustard (*Brassica juncea*, GM) and radish Tango (*Raphanus sativus*, RT) are rich in phenolic compounds, flavonoids, anthocyanins, and isothiocyanates, which display strong antioxidant and antimicrobial activities [20,21]. Extracts from these species have been reported to maintain fruit and meat quality when used as natural preservatives [22,23]. Recent work from our group has shown that an alginate-based coating containing GM and RT sprout extracts can extend tomato shelf life by up to 30 days [24]. As sprouts often accumulate 2–10 times more phytochemicals than mature plant tissues, they represent a potent and underexplored source of natural antioxidants for active packaging [25]. Building on these insights, the present study explores Brassica juncea and Raphanus sativus sprout extracts as active agents for edible coatings with combined antioxidant and antimicrobial properties. The extracts were encapsulated within zein/chitosan (Z/CH) microparticles prepared by complex coacervation and subsequently integrated into PVA and chitosan matrices to form composite films. A key design consideration in this study is that zein/chitosan (Z/CH) microparticles do not possess sufficient film-forming ability to generate free-standing coatings on their own and therefore must be incorporated into an external dispersion (continuous) phase. In addition, direct incorporation of sprout extracts into film-forming polymers is limited by phase incompatibility, poor stability during processing, and uncontrolled release. Encapsulation of the extracts into Z/CH microparticles was therefore employed to create a stable intermediate phase that bridges the physicochemical mismatch between the bioactive compounds and the hydrophilic polymer matrices. On this basis, polyvinyl alcohol (PVA) and chitosan were selected as two distinct dispersion matrices to test how the chemistry of the continuous phase governs microparticle dispersion, interfacial interactions, and functional performance. Evaluating these matrices separately enables isolation of matrix-driven effects prior to blending optimization. The work introduces a dual biopolymer encapsulation-film system that enhances stability, mechanical integrity, and sustained release of bioactives. Structural and functional performance were evaluated using FTIR, UV–Vis, TGA, SEM, and contact angle analyses, while antioxidant activity was monitored throughout the processing. To our knowledge, this is the first report to describe the encapsulation of sprout extracts in Z/CH microparticles for biodegradable, edible coatings, providing a scalable and natural approach to food preservation. Although the encapsulation of plant-derived extracts has been widely explored, most studies focus on generic extracts, single-polymer carriers, or delivery systems not designed for integration into solid edible films. In contrast, the present work specifically targets bioactive-rich sprouts of *Raphanus sativus* and *Brassica juncea*, which are characterized by a high content of reactive phenolics and isothiocyanates but suffer from limited stability during processing and film formation. A zein/chitosan complex coacervate was deliberately selected to address these challenges by combining the hydrophobic affinity of zein for sprout-derived compounds with the film-forming ability and intrinsic antimicrobial properties of chitosan. Importantly, this study does not treat encapsulation as an endpoint, but rather as a functional strategy to enable the incorporation of unstable natural actives into processable, castable edible films, while preserving their antioxidant and antimicrobial performance under high-shear and drying conditions. To the best of our knowledge, this is the first report to demonstrate the integration of sprout-derived bioactives encapsulated in zein/chitosan coacervates into biodegradable polymer films retaining functionality.

## 2. Materials and Methods

### 2.1. Materials

Zein (Z), chitosan high molecular weight (CH_HMW_) (310–375 kDa and deacetylation degree 75%), chitosan low molecular weight (CH_LMW_) (150–500 kDa, and deacetylation degree 75%) (CH_HMW_: catalog no. 419419; CH_LMW_: catalog no. 448869). Tween 20 (T20), polyvinyl alcohol (PVA) (molecular weight 200 kDa), pectin (P) from apples (10 to 300 kDa) with the Degree of Esterification of 50–75% and galacturonic acid of 75–80%. The molecular weight ranges of the polymers were provided by the manufacturer and were not determined experimentally in this study. Ethanol and acetic acid were all purchased from Sigma-Aldrich (Milan, Italy). Ethanolic solution of 2,2-diphenyl-1-picrylhydrazyl (DPPH) from Sigma-Aldrich (Milan, Italy).

Low-molecular-weight chitosan (CH_LMW_) was used for the preparation of zein/chitosan microparticles, whereas high-molecular-weight chitosan (CH_HMW_) was employed for film formation due to its superior film-forming ability and enhanced mechanical stability.

### 2.2. Green Mustard (GM) and Radish var. Tango (RT) Extraction and Characterization

Seeds of Brassica juncea L. (green mustard, GM) and Raphanus sativus var. Tango (radish, RT) was obtained from Italian Sprout (Cesena, Italy) and germinated for five days in the dark at 22 °C. The resulting sprouts were rapidly frozen in liquid nitrogen, finely powdered, and lyophilized at 1 °C under 9.5 × 10^−2^ mbar (LIO 20 FP, 5Pascal, Milan, Italy). Lyophilized GM (2.12 g) and RT (2.36 g) powders were extracted in 80% ethanol using 250 mL and 280 mL of solvent, respectively. Each suspension was sonicated for 30 min (XUBA3, Grant, Swindon, UK) and stirred overnight at room temperature. The filtrates were concentrated under reduced pressure at 50 °C and 80 mbar (IKA RV8, Staufen, Germany), and the residual aqueous phases were freeze-dried (Alpha 3–4 LSCbasic, Christ, Berlin, Germany). The extraction yields were approximately 24% for GM and 26% for RT. The dried extracts were stored at −20 °C until use. For characterization, extracts were re-dissolved (2 mg mL^−1^) and evaluated for antioxidant activity (AA), total phenolic content (TPC), and total flavonoid content (TFC). TPC was quantified by the Folin–Ciocalteu method [26], and the results were expressed as µg gallic acid equivalents per mg of extract. TFC was measured according to Pekal and Pyrzynka, using catechin as the standard, and reported as µg catechin equivalents per mg^−1^ extract. The radical-scavenging capacity was determined using the DPPH assay. 50 µL of each extract was mixed with 2.95 mL of ethanolic DPPH (101.4 µM), incubated for 30 min at room temperature, and the decrease in absorbance at 515 nm was monitored using a UV–Vis spectrophotometer (Jasco V-730, Cremella, Italy). Antioxidant activity was expressed as percent inhibition of DPPH radicals.

### 2.3. Preparation of RT and GM Extract-Loaded Microparticles

**Figure 1:**
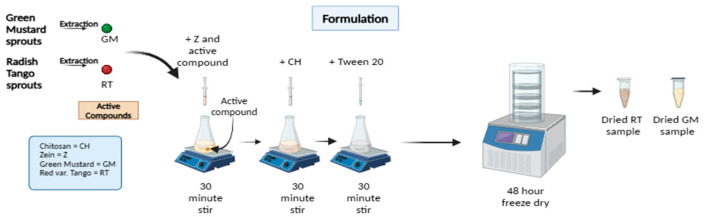
Schematics of the protocol were used to formulate zein/chitosan microparticles containing RT and GM, Created in BioRender. Moeini, A. (2026) https://BioRender.com/l16jf3f.

The encapsulation protocol was refined from previously reported methods [27] by adjusting the polymer-to-polymer ratio, surfactant level, and active-extract concentration to optimize microparticle formation and matrix integration (Figure 1). High-molecular-weight chitosan (CH_LMW_, 0.5% (*w*/*v*) was dispersed in 1% (*v*/*v*) acetic acid and stirred for 24 h at room temperature until a homogeneous solution was obtained. The solution was subsequently filtered under vacuum to remove any undissolved material prior to use.

Separately, zein (Z) solutions at 1% and 2% (*w*/*v*) were prepared in 75% (*v*/*v*) ethanol. Tween 20 (T20) was introduced as a non-ionic surfactant to promote emulsification and droplet stabilization. Stock T20 solutions (5% and 10% *v*/*v*) were briefly vortexed (approximately 40 s) before use to ensure uniform mixing.

For each formulation, the RT or GM extract was dispersed in the zein phase to achieve final concentrations of 4%, 6%, or 8% (*w*/*w*) relative to zein, and the mixture was gently stirred for 30 min at room temperature to promote preliminary association between the extract and the protein. The chitosan solution was subsequently added dropwise while stirring to achieve Z:CH ratios of 5:1 and 7:1. After an additional 30 min of mixing, a defined volume of Tween 20 was incorporated to ensure complete surfactant distribution, followed by a further 30 min of agitation.

The resulting dispersions were designated Z/CH/RTx or Z/CH/GMx, where x denotes the extract concentration (e.g., RT6 for 6% *w*/*v* radish extract; see Table S2). Microparticles were generated through spontaneous complex coacervation between the ethanolic zein phase and the aqueous chitosan phase, in the presence of a surfactant, producing uniform particles without the need for external cross-linking agents. The dispersions were subsequently dried in a vacuum oven at 50 °C and stored until further characterization.

### 2.4. Edible Coating Formulation: MPs in PVA, Pectin, and CH, Polymer Matrices

Blank or extract-loaded microparticles (RT or GM) were incorporated into polymeric matrices composed of high-molecular-weight chitosan (CH_HMW_), polyvinyl alcohol (PVA), and pectin (P). These polymers were initially evaluated as dispersing and film-forming phases in the preparation of edible coatings. Solutions of PVA and pectin (1% *w*/*v*) were prepared in distilled water, while CH_HMW_ (1% *w*/*v*) was dissolved in 1% (*v*/*v*) acetic acid under constant stirring until complete solubilization. The PVA and chitosan solutions were subsequently filtered under vacuum to remove undissolved residues. To enhance the interfacial compatibility between the hydrophobic microparticles and the hydrophilic polymer matrices, Tween 20 (T20) was incorporated at concentrations of 5%, 8%, or 10% (*w*/*v*). At these concentrations, T20 acts as a compatibilizing agent and may also contribute to plasticization of the resulting films. The resulting dispersions were stirred for 3 h to ensure homogeneity. The pre-formed Z/CH/RTx, Z/CH/GMx, and blank microparticle suspensions were then individually added to the polymer solutions and homogenized using an Ultra-Turrax T50 (Ika-Werke, Staufen im Breisgau, Germany) at 10,000 rpm for 5 min in an ice bath. This procedure facilitated uniform dispersion while minimizing structural disruption of the microparticles. The mixtures were further stirred overnight to ensure complete integration of the particles within the polymer matrices. Different polymer-to-MPs volume ratios (3:1, 2:1, and 1:1) were examined to assess the influence of microparticle loading and shear intensity during homogenization on the overall film morphology and stability.

The coating formulations obtained were cast and air-dried under a fume hood at ambient temperature (23 ± 2 °C, 50 ± 5% RH) for 48 h to achieve equilibrium moisture content. The dried films were stored in desiccators until subjected to physicochemical characterization.

### 2.5. Physicochemical Characterizations

#### 2.5.1. Fourier-Transform Infrared Spectroscopy (FTIR) and UV-Vis Spectroscopy

Attenuated total reflection Fourier-transform Infrared spectroscopy (FTIR) was conducted using a Perkin-Elmer Spectrum 100 Spectrometer (Waltham, MA, USA) with a Universal ATR diamond crystal-sampling accessory. The examination was carried out at room temperature. Spectra were acquired by averaging 64 scans (480–4000 cm^−1^), with a resolution of 4 cm^−1^. No smoothing or other mathematical adjustments were used. Spectroscopic manipulations, including baseline adjustment and normalization, were carried out using the Spectracalc software package OMNIC 9 (Thermo Fisher Scientific, Munich, Germany). The collected data were graphed and analyzed using Microsoft Excel 2021. A PerkinElmer LAMBDA 850+ UV/Vis Spectrophotometer (Waltham, MA, USA) was used to perform UV-vis spectroscopy.

#### 2.5.2. Loading Efficiency

The PerkinElmer LAMBDA 850+ UV/Vis Spectrophotometer was used to define the loading efficiency (LE) of the GM and RT extract into the MPs. To construct calibration curves of both active compounds (ACs), RT and GM extracts were diluted with ethanol (75% *v*/*v*). The calibration curves were generated using 10 dilution points ranging from 4 to 0.14 mg mL^−1^ for GM and from 2.3 to 0.06 mg mL^−1^ for RT (Figure S1). The average peak absorbance value at each concentration was calculated from triplicate spectra. The data were then fitted with linear regression functions: y = 0.271x − 0.0241 (R2 = 0.9954) for GM and y = 0.4961x − 0.0134 (R2 = 0.9879) for RT. Well-defined peaks, free from excessive data noise, confirmed the method’s accuracy, as shown in Figure S2. To determine the encapsulation efficiency of RT and GM in Z/CH MPs, the samples listed in Table S2 were analyzed as described. For LE analysis, dried encapsulated microparticles were dispersed in 75% (*v*/*v*) ethanol to completely solubilize the zein wall, ensuring full release of polyphenolic compounds and accurate loading efficiency, and placed on an orbital shaker at medium speed for 30 min, and taken off right before the UV measurement to ensure the solutions remain homogenous. The orbital shaker was used solely for loading efficiency analysis, facilitating the diffusion of encapsulated compounds into ethanol without mechanically disrupting the microparticles. This step was distinct from the film formulation process.

The following equation (Equation (1)) and the individual linear regression slopes obtained from the calibration curves were used to determine the loading percentage of samples prepared at varying concentrations. The loading efficiency values of the samples are reported in Table S2.

(1) $$LE(\%)=\frac{Amount\;of\;compound\;loaded}{Total\;amount\;of\;compound\;initially\;used}\times 100$$

#### 2.5.3. Thermogravimetric Analysis (TGA)

Thermogravimetric analysis (TGA) was performed using a PerkinElmer Pyris Diamond TG-DTA (Waltham, MA, USA) with 7 ± 2 mg samples in a nitrogen atmosphere (flow rate: 30 mL min^−1^). The thermal program was 30–100 °C (20 °C min^−1^), followed by a 30 min isotherm, and then heating to 700 °C (10 °C min^−1^). The temperature that corresponded to the 5% weight loss in the TG curves was determined to be the onset degradation temperature (T_onset_). The temperature that corresponded to the maximum peak in the derivative thermogravimetric (DTG) curve was determined to be the temperature of the maximum degradation rate (T_max_). The char value was calculated as the residual weight at 600 °C.

#### 2.5.4. Mechanical Properties

Tensile tests were performed on films using an Instron model 5564 dynamometer (Pianezza, Italy) equipped with a 1 kN load cell at 23 ± 2 °C, 45 ± 5% RH, with a 5 mm min^−1^ clamp separation rate. The experimental data are an average of 3 determinations.

#### 2.5.5. Scanning Electron Microscopy (SEM)

A JEOL JSM-7200F scanning electron microscope was used for SEM, while a white light optical microscope was used for microscopic examinations. Gold sputtering was performed on the samples in a Hummer JR vacuum chamber after they were secured to the specimen holder using double-sided copper tape. The specimen holder was then inserted into the scanning electron microscope, and ImageJ 1.54i software was used to further process and analyze the images to examine and estimate particle size. In a subsequent analysis, a FEI Quanta 200 FEG microscope (FEI Company, Hillsboro, OR, USA) was used to perform scanning electron microscopy on cryofractured sample cross-sections. After coating the samples with an Au/Pd layer, secondary electron detection and an accelerating voltage of 30 kV were used to obtain SEM images.

#### 2.5.6. Water Contact Angle

A DSA 25 optical contact angle analyzer (KRÜSS GmbH, Hamburg, Germany) equipped with a high-resolution camera was used to evaluate the wettability of the films. The KRÜSS ADVANCE software was used to analyze the results. To determine the static contact angle of room-temperature water using the sessile drop method, ten drops of pure water (2 μL) were placed onto the surface film using a syringe. The entire droplet was released from 0.5 cm above the polymer surface to ensure consistent measurements. The contact angle was measured immediately after droplet deposition by determining the angle between the drop’s baseline and its tangent. The boundary average angle values were then calculated for all the drops. After calibrating the software to the camera, a mechanical syringe is activated from the software, releasing a droplet on each film placed beneath it on a metal plate. After the drop was discharged, the camera took a picture of the drop and analyzed the left and right angles from the droplet’s known distance and the syringe’s size. Three different areas of each film sample were measured in this manner, and the average contact angle was calculated in Microsoft Excel 2021.

### 2.6. Biological Assay

#### 2.6.1. Antioxidant Assay of MPs and Films and In Vitro Release

The antioxidant performance of RT and GM extracts, as well as the corresponding loaded and unloaded microparticles and films containing or lacking MPs, was evaluated using the DPPH radical-scavenging method, as described by Moccia et al. (2020) with minor adjustments [28]. Approximately 4 mg of each MP sample was immersed in 3 mL of an ethanolic DPPH solution (101.4 µM) inside a cuvette. Absorbance changes were recorded at 515 nm using a Jasco V-730 UV–Vis spectrophotometer over multiple intervals (30 min, 1, 2, 4, 6, 24, 48, 72, 96, and 168 h) and denoted as A_1_. A blank sample containing DPPH without MPs (A_0_) served as the control for every measurement. For films, 1 cm^2^ sections were weighed and analyzed under the same conditions. The radical-scavenging ability was calculated as a percentage inhibition of DPPH per mg of film according to Equation (2). All measurements were conducted in triplicate.

DPPH inhibition (%) = ([(A_0_ − A_1_)/A_0_])/(film (mg)) × 100 (2)

#### 2.6.2. Antimicrobial Test

The antimicrobial activity of the coatings was assessed against *Salmonella enterica* serovar Typhimurium (ATCC 19585). The strain was cultured in Tryptic Soy Broth (TSB) for 18 h at 37 °C under shaking (200 rpm), then diluted in 0.8% NaCl to approximately 5 × 10^5^ CFU mL^−1^.

Circular coupons (radius 1.5 cm) were prepared by cutting dried polymer films containing GM or RT into disks and inoculated with 20 µL of bacterial suspension. Polyethylene terephthalate (PET) and uncoated polymer films (chitosan and PVA) served as controls. Samples were incubated at 4 °C for 24 h to simulate refrigerated contamination. After incubation, each coupon was transferred into 1 mL of phosphate-buffered saline (PBS), vortexed for 60 s, and 50 µL aliquots were plated on XLD agar to enumerate surviving colonies. Six replicates were analyzed, each using an independent coupon as a biological replicate.

An additional agar-diffusion assay was performed. The same bacterial suspension, adjusted to 0.5 McFarland turbidity, was spread (100 µL) on tryptic soy agar (TSA) plates. Antibiotic disks containing 20 µL of 5 mg mL^−1^ tetracycline served as positive controls. Disks (0.5 cm diameter) of each coating were placed on separate plates, one formulation per plate, and incubated at 37 °C for 18 h. Inhibition zone diameters were measured with a digital caliper.

### 2.7. Statistical Analysis

Peak numbers in FTIR spectra were calculated using the Origin Pro 2023 software (Origin-Lab Corporation, Northampton, MA, USA) peak analyzer. The loading efficiency and contact angle values were calculated using Microsoft Excel 2021, with means and standard deviations (Microsoft Corporation, Redmond, WA, USA, 2018). All experimental data were evaluated using a one-way analysis of variance (ANOVA) in R (version 4.3.2 for Windows) with a significance level of *p* > 0.05. Tukey’s Honest Significant Difference (HSD) test assessed significant differences among the means. The statistical analysis of the microbiological part was conducted using nonparametric comparisons for each pair, as implemented in the Wilcoxon method within the JMP (SAS) package. Excel was used only for descriptive statistics and data aggregation, while inferential statistics were performed in R (ANOVA/Tukey), and JMP was used exclusively for the microbiological non-parametric comparisons (Table 1).

## 3. Results and Discussion

Encapsulation of sprout extracts into zein/chitosan microparticles offers several advantages over the direct incorporation of free extracts into film-forming solutions. First, the extracts contain phenolics and isothiocyanates that are poorly compatible with hydrophilic polymer matrices, leading to phase separation, aggregation, and non-uniform distribution when added directly. Encapsulation creates an intermediate carrier phase that improves dispersion within the continuous matrix and protects sensitive compounds during high-shear processing and film drying. Second, the protein–polysaccharide microparticle structure promotes stronger interfacial interactions with the surrounding film, reducing rapid migration and enabling contact-active or sustained effects rather than uncontrolled release. Third, encapsulation mitigates the negative impacts of free extracts on film integrity, such as brittleness or excessive plasticization, thereby preserving mechanical and handling properties. Preliminary formulation trials involving the direct addition of free extracts to the polymer solutions resulted in poor homogeneity and unstable films, and were therefore not pursued further. For these reasons, encapsulation was adopted as a necessary formulation strategy rather than an optional modification.

### 3.1. Characterization of GM and RT Extracts

The extractive yield, total phenolic content (TPC), total flavonoid content (TFC), and antioxidant activity (AA) of the RT and GM sprout extracts are listed in Table S3. While there were no appreciable variations in the TFC, the extractive yield and TPC of RT were greater than those of GM. Furthermore, the RT values of AA of the extracts as determined by the DPPH assay were significantly greater than those of GM (16.5% inhibition of DPPH vs. 9.3% inhibition of DPPH). This implies that RT is superior to GM at scavenging free radicals. Over 168 h, the AA of the two extracts was also assessed (Figure S3). The antioxidant capacity increased rapidly during the initial 6 h for both extracts, after which a gradual deceleration continued until 168 h. The difference in antioxidant activity between the two extracts remained consistently around 50% from 24 to 168 h.

### 3.2. Preparation and Characterization of RT and GM-Loaded Microparticles

#### 3.2.1. Fourier-Transform Infrared Spectroscopy (FTIR), UV-Vis Spectroscopy, and Loading Efficiency

The physical or chemical interfaces between components in polymer-based systems can be resolved using FTIR. Therefore, FTIR was used to characterize MPs, qualitatively identifying the encapsulated RT and GM in Z/CH MPs, as well as the interaction of the ACs with the CH and Z matrices. The key FTIR peaks and their corresponding functional group assignments for zein, chitosan, the encapsulated microparticles, and formulated films are summarized in Table S4 to facilitate spectral interpretation and comparison.

**Figure 2:**
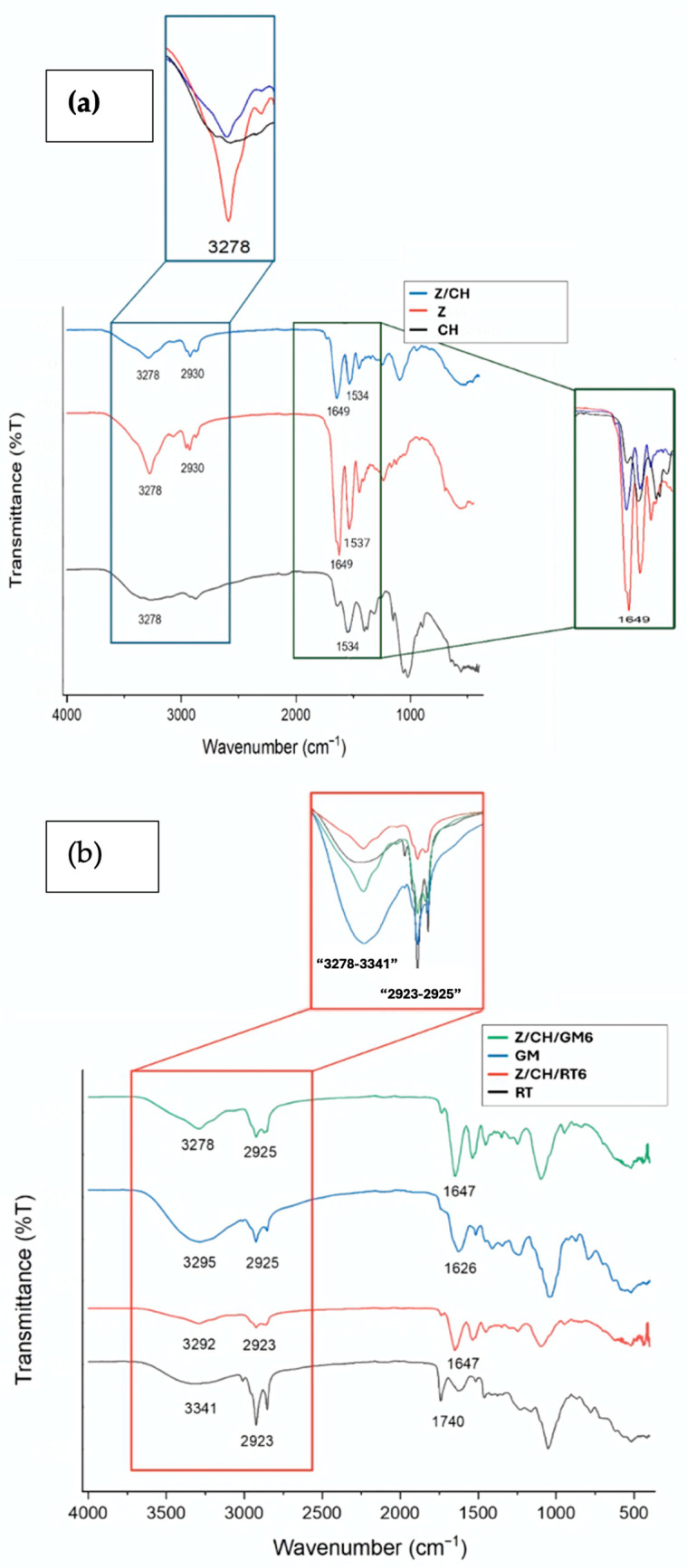
(**a**) Assessment of the FTIR spectra of zein (Z) and chitosan (CH) with blank zein/chitosan (Z/CH) MPs with a ratio of 5:1. (**b**) Comparison of the FTIR spectra of neat Red Tango (RT) and Green Mustard (GM) with zein/chitosan (Z/CH) samples, ratio 5:1, formulated with 30 mg RT and GM microparticles.

As Figure 2a shows, in the zein spectrum, a relatively sharp peak at 3289 cm^−1^ accounted for the stretching vibrations of the amino acids’ O–H and N–H bonds, while the peaks around 2930 cm^−1^ indicated C–H groups [29]. The 1645 cm^−1^ peak was ascribed to the C=O bond stretching in the carbonyl groups of amides (amide I) [30]. The peak at 1537 cm^−1^ (amide II) is attributed to the N–H bending, and the C–N stretching is located [29]. The band observed at 1240 cm^−1^ corresponds to the axial stretching of C–N bonds and C=O bending vibrations (amide III), reflecting the presence of amino acid structures [27]. Pure chitosan exhibited two characteristic peaks at 1653 cm^−1^ and 1534 cm^−1^, ascribed to N-acetylglucosamine residues’ C=O stretching (amide I) and N–H bending (amide II) vibrations, respectively [31].

In Z/CH MPs, the three main peaks of Z at 3278, 2930, and 1649 cm^−1^, as well as the peak at 1534 cm^−1^ of CH, provide evidence of the existence of both Z and CH in the Z/CH MPs (Figure 2a) [32,33]. The hydrogen bonding interaction between zein and chitosan could be evidenced by the widening of the peak at 3278 cm^−1^, while the formation of the small peak at 1740 cm^−1^ likely results from the presence of T20 or the interaction of zein and chitosan [34].

The first peak of interest in the RT spectra is the OH group peak, located at 3341 cm^−1^ (Figure 2b). The widening of the peaks in the encapsulated samples is attributed to hydroxyl stretching vibrations [34].

The wavenumber of interest displayed is 2925–2923 cm^−1^ across all samples, with slight variation between spectra (Figure 2b). A peak at this wavelength suggests the presence of C-H bonding, attributed to the aliphatic C-H stretch in RT and GM, characteristic of encapsulated ACs in Z/CH MPs [35].

The spectra in Figure 2b also display peaks at 1626 and 1740 cm^−1^. Phenolic compounds found in plants typically exhibit a peak close to 1600 cm^−1^ [36]. The shift at higher wavenumber can be due to aromatic and olefinic C=C stretching modes [37], while the peak at 1740 cm^−1^ indicates the presence of carbonyl C=O species in RT.

In the RT-loaded samples, a modest shift of the amide I band from 1649 cm^−1^ to 1647 cm^−1^ was observed compared to the blank Z/CH microparticles. Although subtle, shifts of this magnitude can reflect slight changes in hydrogen-bonding states or protein secondary structure upon interaction with bioactive compounds [38,39]. Similar observations have been attributed to alterations in conformational dynamics or molecular interactions, even when the shifts are only 1–2 cm^−1^ [40]. While such small shifts alone cannot definitively confirm strong binding, together with the loading efficiency results and preserved antioxidant activity, they support the conclusion that RT constituents remain associated with microparticles.

For GM-loaded microparticles, the FTIR spectra exhibited analogous trends, with the O–H stretching band appearing at 3295 cm^−1^. This peak broadened upon encapsulation, suggesting hydrogen bonding interactions between GM phenolic hydroxyl groups and the chitosan or zein matrix. In the carbonyl region, peaks between 1644 and 1651 cm^−1^ correspond to the amide I vibration of zein, with slight shifts from the blank Z/CH spectrum indicating molecular interactions with phenolic compounds.

UV-Vis spectroscopy is a valuable tool for confirming the encapsulation of RT and GM by quantifying the concentration of specific substances before and after their incorporation into the film matrix. The UV-Vis spectrum of Z/CH/RT6 MPs, with the lank MPs dispersion used as a reference, was used to better visualize the absorption of RT and GM peaks in the encapsulated MPs. The successful incorporation of RT and GM into microparticles was confirmed by the shift of the main peaks of GM and RT from 331 nm and 333 nm to 353 nm and 385 nm, respectively, in the encapsulated microparticles (Figure S2). This shift can be attributed to the interaction between polymer matrices and ACs, including hydrogen bonding and changes in the local dielectric environment, which alter the electronic transitions of the encapsulated compounds. This is consistent with the FTIR data [41].

As shown in Table S2, the Z/CH ratio and extract concentration (*w*/*v*%) significantly influenced the loading efficiency (LE) of the MPs (*p* > 0.05). The highest LE observed was 90% for the Z/CH 7:1 RT4 formulation, while the lowest was 27.8% for the Z/CH 5:1 RT4 formulation. A general trend emerged in which higher Z proportions correlated with increased LE, likely due to enhanced hydrophobic domains in the Z-rich matrix, which improved extract incorporation through favorable interactions, such as hydrophobic bonding, or reduced phase separation.

#### 3.2.2. Antioxidant Activity of Sprout Extracts and MPs

The AA of the different extract concentrations loaded into the microparticles was determined; it increased rapidly within the first 24 h and continued to rise more gradually over the subsequent days (Figure S3). At all tested concentrations, the RT extract consistently exhibited greater activity than the GM extract, with a dose-dependent trend. After 24 h, the RT extract achieved 52.0% inhibition, whereas the GM extract reached only 39.4%. By the end of the study period, the inhibition observed for the RT extract further increased to 71.0%, compared to 56.5% for the GM extract (Figure S3). These findings suggest that the RT extract not only acts more rapidly but also achieves a higher overall level of antioxidant inhibition compared to the GM extract. The dose-dependent trend observed in both extracts reinforces the influence of extract concentration on the overall activity.

**Figure 3:**
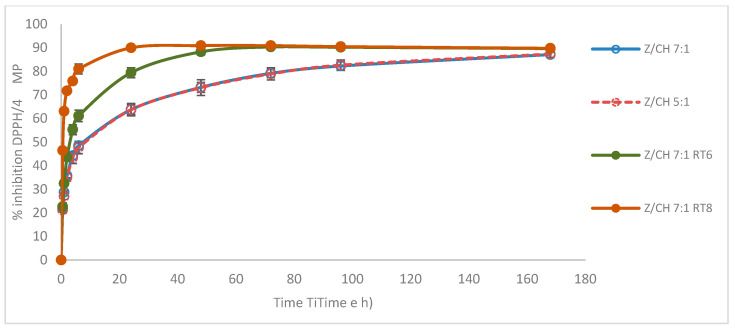
Antioxidant activity of 4 mg unloaded MPs and Z/CH 7:1 loaded with RT6 and RT8 over 168 h.

The AA measured during 168 h was similar for the unloaded MPs, Z/CH 7:1 and Z/CH 5:1, consistently lower than the AA exhibited by the loaded MPs (Figure 3). In particular, the Z/CH 7:1 RT8 sample reached 90% DPPH inhibition after 24 h and remained stable as all the substrate (DPPH) was consumed, whereas the lower concentration (RT6) showed a 90% DPPH inhibition after 72 h (Figure 3). All the other loaded MPs reached 90% after two to four days (Figure S4). The major differences between unloaded and loaded MPs were recorded during the first hour, and after 48 h, the differences were about 40%. Notably, the uncharged MPs exhibited antioxidant properties over a period of one week. Indeed, zein has been reported to possess antioxidant features due to the presence of antioxidant amino acids [42]. However, the unloaded MPs displayed slower kinetics, indicating that zein alone exerts a weaker antioxidant effect than when combined with the extract. Thus, combining zein MPs with the extracts leads to a more pronounced effect.

The comparison between AA in free extracts and in encapsulated MPs shows that, while both free extracts exhibit significant activity, encapsulation enhances stability and allows for more controlled, prolonged release, particularly for the RT extract.

### 3.3. Edible Coating Active Film Characterizations

#### 3.3.1. Formulation of Active PVA, P, and CH Films

Once the MPs’ functionalities were assessed, the most suitable biodegradable polymers with potential compatibility with the MPs and film-forming ability were investigated to prepare edible coating formulations. Because Z/CH microparticles lack intrinsic film-forming capability, all formulations required an external dispersion phase to obtain mechanically stable films. Films were prepared using PVA, CH_HMW_, and P from apples, in varying proportions, serving as the dispersing phase (DP) and thickening agents for the MPs. In Petri dishes, different ratios of microparticles (MPs) and biopolymers were dispersed to create films. The suspensions were homogenized using an Ultra-Turrax at 10,000 rpm in order to reduce agglomeration and ensure uniform dispersion, as magnetic stirring during initial preparation led to particle aggregation. While this high-shear treatment may have partially disrupted some MPs, it proved to be the most effective method for achieving uniform distribution prior to casting. Overall, the process yielded a well-dispersed polymer matrix with preserved particle integrity and a narrow size distribution (see Section 3.3.4).

Figure S5 shows the cast films after drying. It was observed that pectin (P) was not a viable dispersion phase for the MPs due to insufficient film-forming ability and poor compatibility after formulation. Accordingly, pectin-based formulations were excluded from further investigation. Both PVA- and CH-based films were homogeneous, with very smooth surfaces in most samples, and the CH film containing a 5:1 (Z:CH) ratio was the most flexible and pliable (Figure S5). The PVA and CH films containing a 7:1 ratio were comparatively less homogeneous (Figure S5).

Finally, based on casting data, the samples containing MPs with a Z:CH ratio of 5:1 best met the homogeneity and plasticity criteria and were selected for further testing. This involved the preparation of PVA and CH-based films formulated with different proportions of the MPs containing 6% *w*/*v* of RT and GM (DP:MPs ratios of 3:1, 2:1, and 1:1), to produce active films potentially used as an edible coating (Figure S6). FT-IR analysis, SEM, TGA, wettability tests, and antioxidant assays were conducted to comprehensively understand the effects of MPs on biofilms. These methods determined the optimal formulation of active edible films by adding sprout extracts as ACs.

#### 3.3.2. Fourier-Transform Infrared Spectroscopy (FTIR)

**Figure 4:**
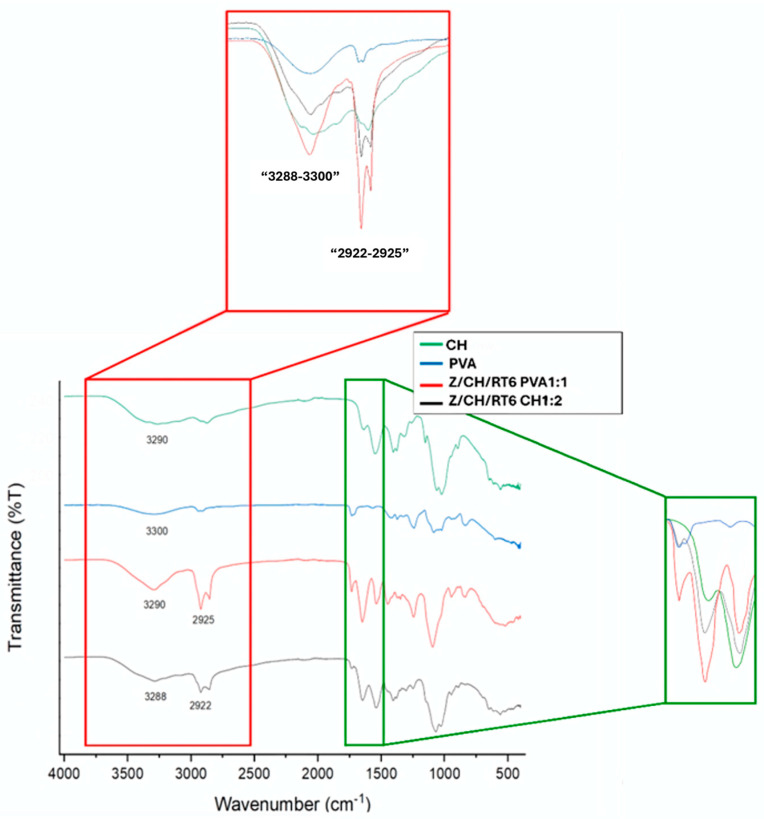
FTIR spectra comparison of CH and PVA films with encapsulated MPs in PVA 1:1 and CH 1:2 ratio for the dispersed phase.

**Figure 5:**
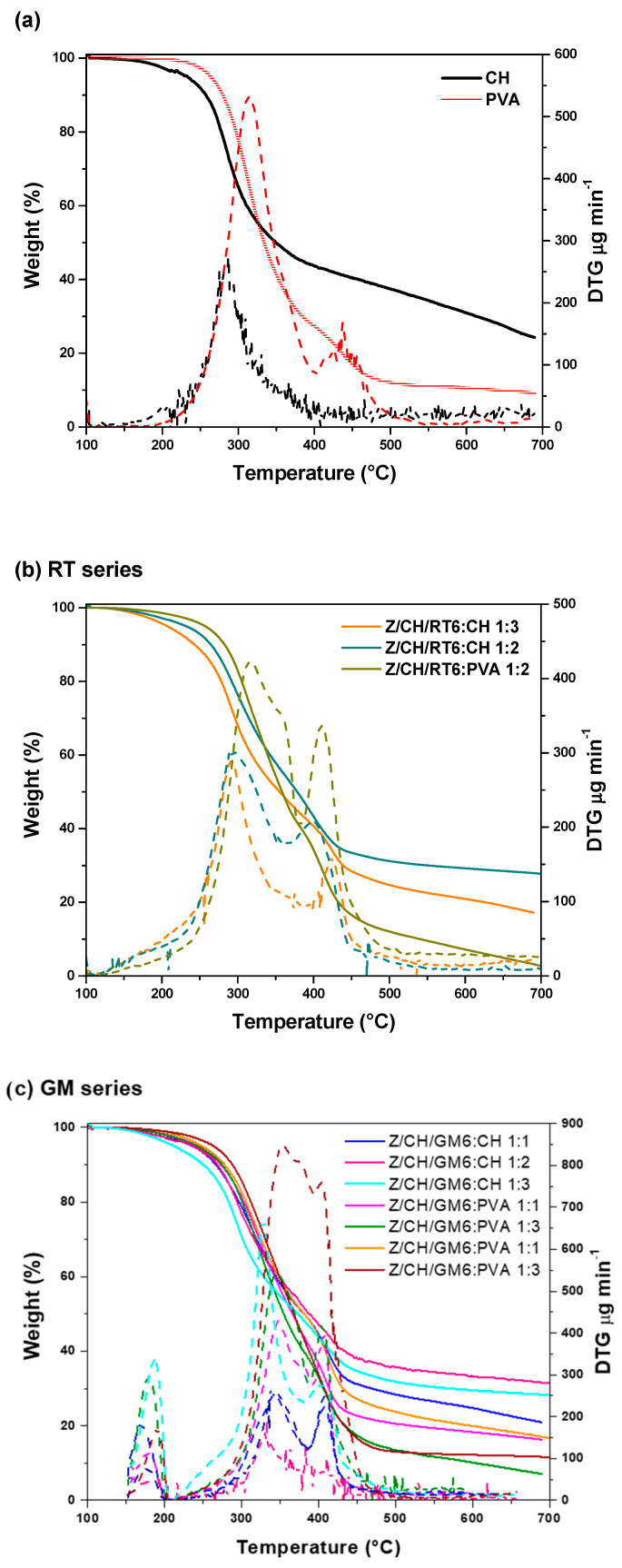
TGA curves of (**a**) PVA and CH films, (**b**) PVA/CH-based films, RT series, and (**c**) PVA/CH-based films, GM series. Full lines refer to thermogravimetric curves, dotted lines to DTG curves.

FTIR spectra of the chitosan (CH) film (Figure 4) showed distinct peaks corresponding to N-acetylglucosamine residues, exhibiting C=O stretching (amide I) and N–H bending (amide II) vibrations. The band near 1653 cm^−1^ may include contributions from both amide I and the asymmetric N–H_3_^+^ deformation [43], while the stronger peak at 1564 cm^−1^ likely results from the overlapping amide II and primary amine vibrations. The greater intensity of this band indicates a higher proportion of amine groups within the polymer matrix. Main peaks related to hydroxyl and acetate groups were observed in PVA (Figure 5). The O-H stretching from the intramolecular and intermolecular hydrogen bonding is responsible for the wide bands seen between 3288 and 3300 cm^−1^ [44]. The vibrational band observed between 2922 and 2925 cm^−1^ refers to the C–H stretching from alkyl groups, and the peaks between 1750 and 1735 cm^−1^ are due to the stretching of C=O and C–O from the residual acetate group [45].

The successful formulation and interaction among encapsulated MPs, PVA, and CH were evidenced by the FTIR spectrum of the formulated films. The broad, more intense peak at 3288–3300 cm^−1^, the shift in peak position between 1600 and 1559 cm^−1^, and the change in intensity, especially in the PVA film, can be attributed to hydrogen bonding between the MPs and the matrices. Additionally, good interaction between PVA and MPs in Z/CH/RT6 PVA 1:1 is hypothesized, as a sharper and more intense peak at 2929 cm^−1^ was observed [46]. Furthermore, a strong peak at 1600 cm^−1^, attributed to the amide groups of Z, suggests that the zein-based MPs were well incorporated into the PVA film via hydrogen bonding [44]. The presence of characteristic peaks of RT and GM extracts in the film spectra does not necessarily indicate complete release, as such signals may result from partial surface exposure of encapsulated compounds, thin polymer shells, or a minor free fraction within the matrix, consistent with previous observations in similar biopolymer–microparticle systems [47,48].

#### 3.3.3. Thermogravimetric Analysis (TGA)

TGA was performed on PVA and CH-based films; the relative TG curves are shown in Figure 5, while the data obtained from the TG curve analysis are reported in Table S5. Due to their hydrophilic nature, both neat polymers showed high humidity absorption (about 21% for CH), which decreased considerably with the incorporation of the MPs (drop to 4.5% for Z/CH/GM6:PVA 1:3). CH displayed a single thermal degradation step, with a T_onset_ around 233 °C, and main degradation peak at 286 °C, related to the random split of the glycosidic bonds and elimination of volatile products [31]. The sample lost around 70% of its mass at 600 °C. This is typical of CH, confirming that the TGA method was suitable for further thermal analysis of the film samples [8]. Three weight-loss regions were noted in the TG curve of pure PVA, mainly due to water release. Adsorbed water molecules poorly interact with the matrix, while additional water molecules are strongly bound to the hydroxyl groups. An initial weight loss region between 50 and 200 °C can be attributed to the loss of the absorbed water molecules that are physically bonded to the PVA polymer, while the region between 200 and 340 °C is related to the elimination of water chemically bound to the polymer matrix [49,50]. The third region, between 340 and 450 °C, corresponds to the decomposition and carbonization of the polymer. The main degradation step (T_max_) occurred at approximately 314 °C, with approximately 9% residue remaining at 600 °C.

The MPs-doped films exhibited a complex, multistep degradation process, similar to that of PVA, but also correlated with the presence of microparticles containing RT and GM extracts. The incorporation of GM extracts increased T_onset_, particularly in CH-based films. The main thermal degradation (T_max_) occurred at higher temperatures across all extracts. There was no clear trend in char yield with the number of MPs in the formulation. Regarding PVA-based films, all films showed a similar T_onset_, corresponding to the PVA proportion in the formulation. The maximum degradation rate occurred at higher temperatures in doped films than in pure PVA. At the end of the measurement, the MPs-doped samples showed higher char yields than neat PVA, except for Z/CH/RT6:PVA 1:2, which was likely due to the presence of zein and chitosan in the MPs. Overall, the T_onset_ and T_max_ shifts indicate an improvement in thermal resistance resulting from the incorporation of zein microparticles. These shifts suggest that the formulated films are more thermally stable, which may be attributed to interactions between the zein microparticles and the polymer matrix, potentially leading to a structure that resists thermal degradation more effectively [30].

#### 3.3.4. Scanning Electron Microscopy (SEM)

SEM analysis was employed to investigate the morphological features, distribution of MPs, and surface properties of the films.

**Figure 6:**
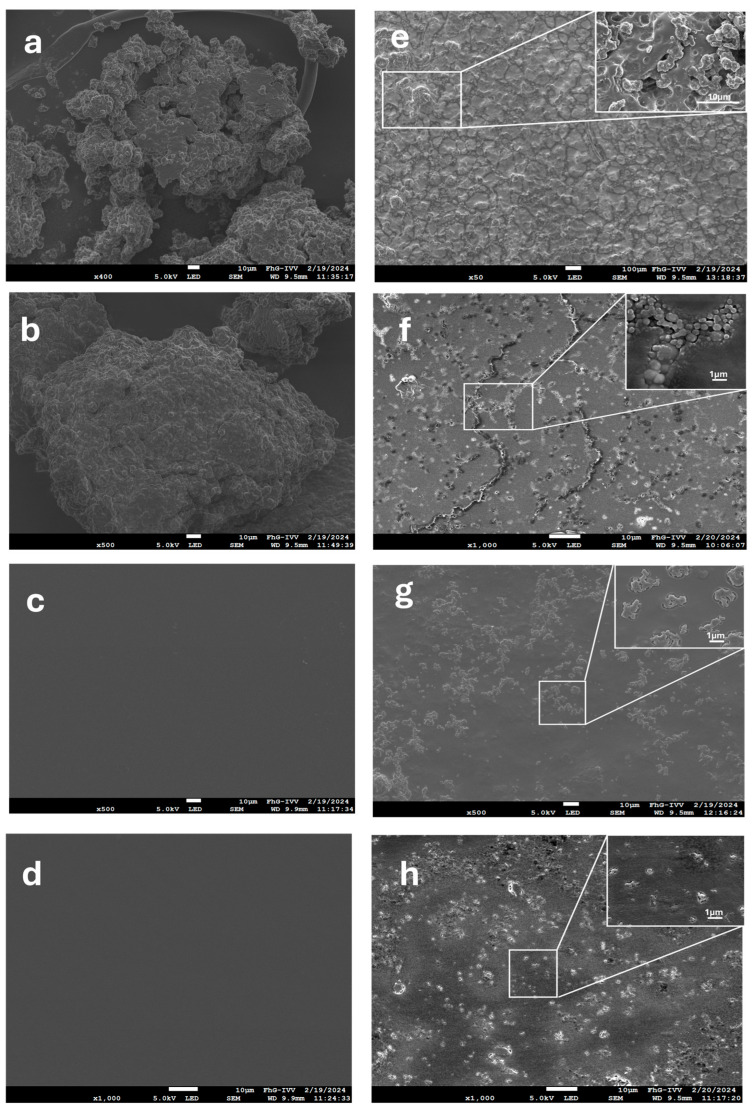
SEM Images of the powder extracts and film surfaces: GM (**a**), RT (**b**), CH (**c**), PVA (**d**), Z/CH/RT6 PVA1:1 (**e**), Z/CH/RT6 PVA1:3 (**f**), Z/CH/RT6 CH1:1 (**g**) Z/CH/RT6 CH1:3 (**h**).

In Figure 6, the micrographs of GM and RT extracts presented bulky granular structures (Figure 6a,b). The neat CH and PVA film surface micrographs exhibit a very even, smooth appearance (Figure 6c,d). The smooth, visually similar surface morphology observed for the neat PVA and chitosan films can be attributed to the solvent-casting process and to the presence of T20, which acts as a plasticizer and compatibilizing agent. At the concentrations used, T20 enhances polymer chain mobility and promotes uniform film formation, leading to dense, homogeneous, and relatively featureless surfaces at the selected magnification. As a result, morphological differences between the two polymers are minimized in the absence of microparticles, whereas more pronounced surface features emerge upon microparticle incorporation, as shown in Figure 6e–h. However, compared with CH films, PVA films showed a rougher but more regular surface, with a more uniform distribution of particles within the film, probably due to better interaction of zein-based MPs with the PVA matrix, as discussed in the Section 3.3.2. It was noted that the particles largely maintained their structural regularity, were well integrated into the films, and formed a very smooth surface (Figure 6e,f). Distinct voids were also visible, suggesting that the microparticles tend to separate or detach more readily from the matrix. In CH films, a clustering distribution of MPs resulted from the inherent properties of the zein-based microparticles and their hydrophobic interactions, which tended to group rather than disperse individually and were then embedded within the chitosan matrix during film formation [47] (Figure 6g,h). Additionally, the particles appeared deformed, and the particle-matrix interface was less defined, indicating stronger interaction or partial embedding of the microparticles within the chitosan. This occurrence may be due to the film-preparation process, as chitosan’s viscosity may promote stronger adhesion at the expense of particle integrity.

Particle size is important in edible films’ appearance, structure, stability, and taste, as it can affect the controlled release behavior of encapsulated ACs [51]. The particle size in the films was measured using ImageJ software (Table 2), and it was found to be within the expected range of approximately 1 µm, consistent with an emulsion system based on complex coacervation [52]. ANOVA analysis did not reveal a statistically significant difference in particle sizes between the films (*p* > 0.05).

More specifically, the mean diameters of MPs in the films (Table 1) ranged from 0.92 to 1.57 µm [48,53]. In PVA-based films, particle sizes were relatively uniform; for example, Z/CH/GM6:PVA 1:1 averaged 10.5 ± 0.13 µm, while Z/CH/RT6:PVA 1:2 averaged 13.5 ± 0.11 µm, reflecting good dispersion and minimal aggregation. CH-based films showed slightly broader distributions and occasional clustering, particularly at higher MPs loadings; for instance, Z/CH/RT6:CH 1:2 averaged 1.50 ± 0.12 µm, while Z/CH/RT6:CH 1:3 reached 1.57 ± 0.16 µm. This trend further suggests that the higher viscosity and stronger interparticle interactions in CH matrices can hinder complete, uniform dispersion during casting, resulting in slightly broader particle-size distributions at certain loadings. In contrast, the narrower distributions in PVA align with its lower viscosity and better particle-matrix compatibility, which facilitates more homogeneous embedding at the sub-micron scale. Similar relationships between particle size distribution, polymer viscosity, and dispersion quality have been reported for protein/polysaccharide composite films [34,54].

SEM observations (Figure S7) of the cryofractured surfaces of the doped films were also performed to gain further insight into the MPs’ distribution across the film cross-section. Two samples were selected based on the higher concentration of the matrix, CH or PVA. The micrographs enable the evaluation of the affinity between the matrix and microparticles. In PVA, microparticles clearly preserved their structure; however, they were not retained with sufficient force during fracture, and clear evidence of voids due to poor interfacial interactions was observed. In the CH-based film, the microparticles are well embedded in the matrix, with the voids from cryofracture less evident. However, particle clustering was more evident.

#### 3.3.5. Mechanical Properties

The thickness and mechanical properties of CH and PVA-based films are reported in Table 2. Because T20 was used at relatively high concentrations, its plasticizing contribution must be considered when interpreting the films’ mechanical properties; therefore, comparisons are discussed within formulations containing the same T20 level to distinguish microparticle-related effects from surfactant-induced plasticization. CH films exhibit poor ductility, with tensile strains ranging from 3% to 22%. A moderate impairment in modulus and stress at break was observed with the addition of MPs, as they acted as stress concentrators, facilitating the film’s failure during tensile testing [8,50]. The PVA films showed greater ductility than CH. Neat PVA showed almost 70% strain at break.

The addition of MPs decreased the modulus and stress at break; however, the material’s deformability improved, particularly at an MPs:CH ratio of 1:2. Despite SEM analysis suggesting poor interface bonding, this improvement can be attributed to the observed homogeneous dispersion of particles. The particles may help distribute stress more evenly across the film, reducing localized stparticle dispersionress concentrations that can lead to premature cracking. This would increase the material’s overall ductility by enabling it to deform more uniformly under stress. Additionally, the microparticles can promote the formation of microvoids or microcracks in the material during deformation. These microvoids can facilitate energy dissipation during mechanical loading, effectively absorbing stress and thereby increasing strain at break [55]. This can improve the material’s overall deformability, even if the interface between particles and the matrix is not ideal.

Compared with other biocomposite films, these films exhibit competitive mechanical properties (e.g., up to 244% elongation) within the edible/active packaging category [56].

#### 3.3.6. Wettability Test

Wettability tests were performed to evaluate the effect of the active MPs formulation on the surface free energy (SFE) and hydrophilicity of the PVA and CH films (Table 3). All films’ contact angles were below 90°, indicating that they were hydrophilic. The contact angles of PVA and CH were 83° and 67°, respectively. The contact angle measurements of CH-based films incorporating Z/CH/RT6 microparticles showed a clear dependence on the MP-to-polymer ratio. Among the samples, Z/CH/RT6:CH (1:2) exhibited the lowest contact angle (44.78°), indicating the highest surface hydrophilicity, followed by the 1:1 (54.22°) and 1:3 (77.80°) formulations. This pattern likely reflects the interplay between hydrophilic chitosan and phenolic groups from the RT extract, as well as the increasing contribution of the more hydrophobic zein phase at lower MP loadings. At the intermediate 1:2 ratio, the surface is likely to expose more hydrophilic functional groups, thereby enhancing water spreading behavior. At the lowest MPs loading (1:3), the film matrix surface is more influenced by the continuous CH phase with less extract exposure, whereas at the highest MPs loading (1:1), partial aggregation or surface roughness from embedded particles can slightly increase hydrophobicity compared to 1:2. Similar composition-dependent trends in wettability have been reported for protein/polysaccharide-based composite films containing encapsulated plant extracts [57,58]. The film’s hydrophilicity significantly increased after formulation with zein-based microparticles. This can be explained by considering the film formation method.

Indeed, the film-casting solvent significantly influences the hydrophilicity and hydrophobicity of the matrices [30]. Deng et al. proposed that during the drying of the cast film, the solvent evaporates from the surface inward, leading to the film solidifying from the inside out [58]. This process induces hydrophilic groups on the outer layer of the films [59]. In this case, the interaction between MPs and PVA, mediated by hydrogen bonding and physical intermolecular networks, may also contribute to the increased hydrophilicity of the formulated films. A one-way ANOVA test revealed a slightly statistically significant difference (*p* > 0.05) in SFE total between the two types of DP (PVA and CH). However, no discernible difference (*p* > 0.05) existed between the impact of the varying ratios of each DP on the SFE. Natural variations in film casting, microparticle dispersion, and moisture sensitivity are probably the cause of the comparatively significant standard deviations seen in some samples, which are common challenges in bio-based film development.

From the findings, it can be concluded that PVA, with a higher overall SFE, might promote wettability due to a more hydrophilic surface, which favors water interaction. This superior wettability could be advantageous for applications requiring good adhesion or compatibility with food with high water content [51,60].

### 3.4. Bioassay

#### 3.4.1. Antioxidant and In Vitro Release Test of Films

**Figure 7:**
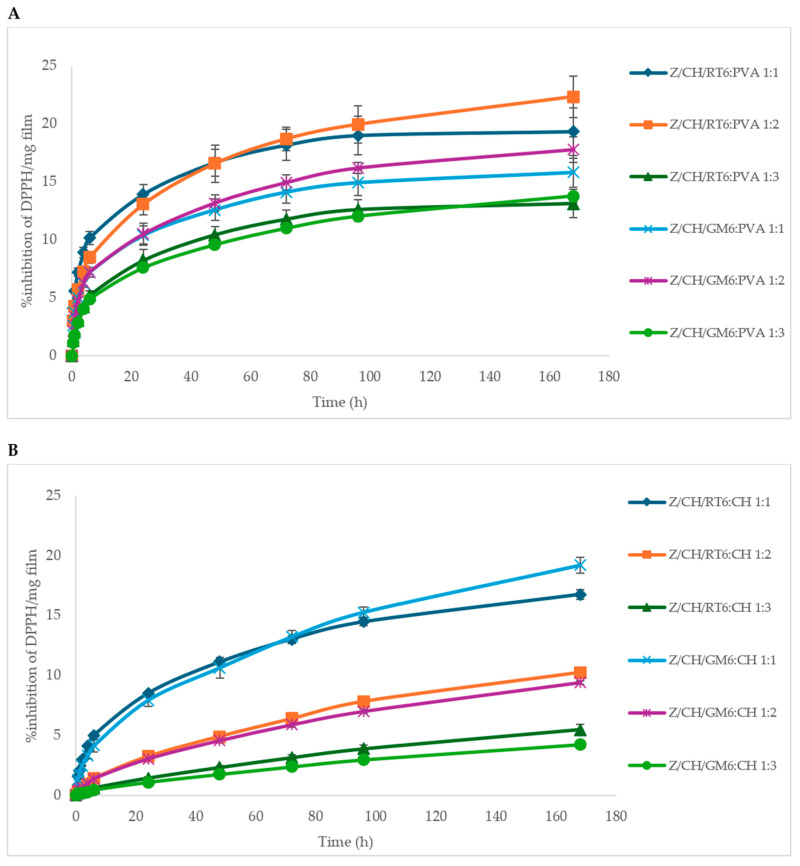
Antioxidant activity of various proportions of Z/CH/RT6 and Z/CH/GM6 MPs formulated into PVA films (**A**) and CH films (**B**).

An antioxidant assay was conducted on blank PVA, CH, and films formulated with various proportions of Z/CH/RT6 and Z/CH/GM6 MPs. The results indicated that the blank PVA and CH showed no antioxidant activity (AA). Figure 7 illustrates the antioxidant activity of Z/CH/RT6 and Z/CH/GM6 MPs formulated in PVA and CH films. Both PVA and CH-formulated films with RT and GM demonstrated consistent AA over time, leading to a gradual increase in antioxidant activity, regardless of the type of film.

In Figure 7A, the antioxidant assay results for different proportions of PVA-formulated film are reported as the percentage of inhibition per milligram of film, due to the varying densities of the films. In general, in both Z/CH/RT6:PVA and Z/CH/GM6:PVA, the films with higher proportions of MPs (1:1 and 1:2) showed higher antioxidant activity than the film with a lower MPs content (1:3). In detail, the highest activity was observed in Z/CH/RT6:PVA1:2 film with 22.4% inhibition, the lowest was 13.2% in the case of Z/CH/RT6:PVA1:3.

The antioxidant activity of different proportions of CH-formulated films is shown in Figure 7B. Also in this case, the AA of Z/CH/RT6:CH and Z/CH/GM6:CH films with a lower MP ratio performed worse than the other formulated proportions, being 5.5% and 4.2%, respectively.

Here, Z/CH/GM6:CH 1:1 and Z/CH/RT6:CH 1:1 MPs exhibited the greatest level of antioxidant activity, with no discernible variations between them up to 96 h. However, by 168 h, the gap increased, and the final inhibition rates were 19.2% and 16.8%, respectively. The other two CH MPs formulated films showed a significantly lower AA than CH 1:1.

When comparing the two best-performing films, PVA:MP/RT 2:1 and CH:MP/GM 1:1, the AA was significantly higher for the first film. In summary, the two best AA have been observed in Z/CH/RT6:PVA (1:2) and Z/CH/GM6:CH (1:1).

It has already been reported that RT leaf extracts used as an active coating were able to preserve the physicochemical properties of sweet lemon fruits [23]. Even GM seed extracts are effective natural ingredients for active packaging [22]. Since radish and mustard sprouts increase the bioactive compound content by 2–10 times compared to mature vegetables or other plant tissues, our extracts could be a good candidate for preparing active packaging with high phytochemical profiles. The sustained antioxidant activity observed in the films is consistent with retention of bioactive functionality after dispersion, although partial release or matrix-mediated protection of non-encapsulated fractions due to the fracture of some MPs cannot be excluded; this interpretation is further supported by the prolonged activity of the microparticles alone (Figure 3).

#### 3.4.2. Antimicrobial Assay Test of Films

**Figure 8:**
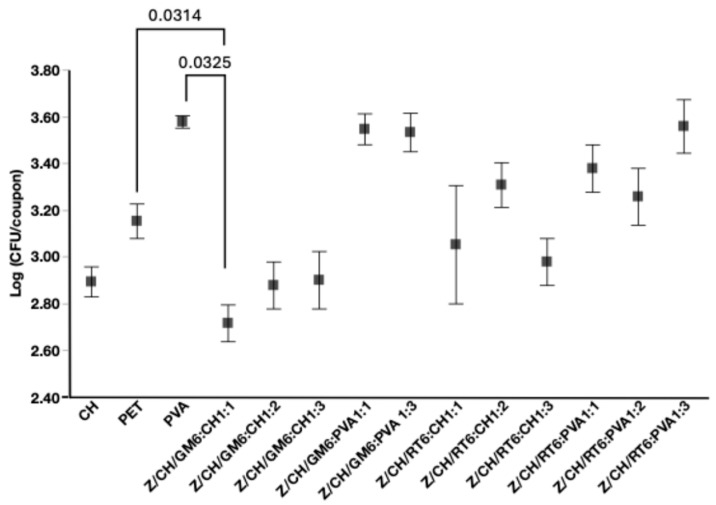
Log reduction of *S*. *typhimurium* cells following 24 h exposure to various coatings. Coating types include green mustard (GM6), red radish (RT6). *S*. *enterica* was tested on chitosan (CH), polyethylene terephthalate (PET), and polyvinyl alcohol (PVA) as a control. Error bars indicate standard error. Horizontal lines represent non-parametric comparisons, with statistical significance indicated by *p* > 0.05.

Chitosan-based films demonstrated measurable antimicrobial activity against *Salmonella enterica*, consistent with its known mechanism of disrupting bacterial cell membranes [31]. In our study, this was confirmed by simulating contact between a contaminated droplet and a chitosan-coated surface. Significant differences in Salmonella reduction were observed among the tested formulations (Figure 8). Control samples, PET and PVA, exhibited higher bacterial loads, with mean Log CFU values of 3.1 ± 0.07 and 3.57 ± 0.02, respectively, indicating no antimicrobial activity. In contrast, chitosan films reduced bacterial counts to 2.8 ± 0.06 Log CFU, confirming their inhibitory effect. Notably, the composite Z/CH/GM6:CH1:1 achieved one of the lowest Log CFU values (2.7 ± 0.07), with minimal variability across replicates. This suggests consistent bacterial suppression and highlights the formulation’s potential as an effective antimicrobial coating, combining the activity of chitosan with enhanced antioxidant properties, as shown in Figure 8. Other promising formulations included Z/CH/GM6:CH1:2 and Z/CH/GM6:CH1:3, which also reduced Salmonella levels (Log CFU 2.87 ± 0.10 and 2.9 ± 0.12, respectively). Similar biocidal effects of chitosan composites have been reported in the literature, particularly when combined with active compounds such as carvacrol and rosemary nanoemulsions [61]. Additionally, the antimicrobial efficacy against *S*. *typhimurium* (Log CFU reduction to ~2.7) is similar to that achieved by chitosan-based coatings containing carvacrol or rosemary nanoemulsions [61], suggesting that the natural sprout extracts encapsulated in Z/CH microparticles are effective alternatives. While those studies were conducted on minced meat, limiting direct comparison, the observed reductions in bacterial load align with our findings from composite screening. In the agar diffusion assay, none of the composite films produced inhibition halos, indicating that antimicrobial effects were likely due to contact rather than the release of diffusible agents. This suggests limited migration of active compounds, which may reduce potential interaction with food matrices. Overall, these findings support the potential of chitosan-based biocomposites, particularly when combined with natural antimicrobials, in developing sustainable active packaging systems designed to control foodborne pathogens and enhance food safety.

### 3.5. Technological Considerations

From a technological perspective, the film preparation approach demonstrated here is highly reproducible and amenable to scale-up. The formulation relies on well-established unit operations, including polymer dissolution, surfactant-assisted dispersion, high-shear homogenization, and solvent casting, which yielded consistent films when repeated under identical conditions. Importantly, these processing steps are compatible with industrial coating technologies, including continuous mixing and roll-to-roll or blade coating processes. While the present study was conducted at laboratory scale, the underlying processing principles are readily transferable to larger-scale production. Future work will focus on pilot-scale validation and process optimization.

## 4. Conclusions

This study successfully demonstrates the encapsulation of novel bioactive extracts from Raphanus sativus (RT) and Brassica juncea (GM) sprouts into zein/chitosan (Z/CH) microparticles (MPs) using a coacervation-based technique, as confirmed by FTIR and UV–Vis analysis. The optimized MPs (Z/CH 5:1 with 6% extract loading) were effectively incorporated into PVA and CH_HMW_ film matrices in various proportions, with homogeneous distribution verified by SEM. Thermal analysis (TGA) indicated that the formulated films exhibited enhanced thermal stability due to the interaction between MPs and the polymer matrices. Despite slight reductions in tensile strength and modulus, the tensile test revealed improved ductility in CH and PVA-based films after incorporating MPs. Contact angle measurements confirmed that all films remained hydrophilic, and the addition of MPs further enhanced surface hydrophilicity, likely due to film-forming dynamics and molecular interactions. Antioxidant assays demonstrated that films with higher MPs content exhibited significant antioxidant activity over time, particularly Z/CH/RT6:PVA (1:2) and Z/CH/GM6:CH (1:1) formulations. In addition, a statistically significant and consistent bacteriostatic effect was observed in Z/CH/GM6:CH (1:1) against Salmonella counts (Log CFU 2.7 ± 0.07), indicating effective bacterial suppression. Although the observed reduction in *Salmonella enterica* counts was below 1 Log CFU, such reductions are commonly classified as bacteriostatic rather than bactericidal and can still be relevant for food preservation by suppressing bacterial growth during storage rather than eliminating pathogens outright. Overall, the results highlight the potential of RT and GM sprout extracts encapsulated in biopolymer microparticles as effective natural additives for developing active packaging materials with enhanced mechanical, thermal, antioxidant, and antimicrobial properties. Future studies should explore the films’ performance in real food packaging applications, optimize bioactive release profiles, and investigate the development of smart, biodegradable packaging systems for broader commercial use.

## Figures and Tables

**Table 1:** Software used for data processing and statistical analysis.

Assay/Dataset	Software (Purpose)
FTIR spectra	OMNIC 9 (baseline correction/normalization); OriginPro (peak extraction)
TGA/DTG (and DSC, if applicable)	Instrument software (data acquisition/export); OriginPro (T_onset_/T_max_/char yield from curves)
SEM image analysis	ImageJ (particle size estimation/image processing)
Loading efficiency (LE) calculations	Microsoft Excel 2021 (calculations; mean ± SD)
Contact angle/wettability	KRÜSS software (angle measurement); Microsoft Excel 2021 (mean ± SD)
ANOVA/post hoc tests (non-microbiological datasets)	R (v4.3.2 for Windows): one-way ANOVA (*p* > 0.05) and Tukey HSD
Microbiological assay statistics	JMP (SAS): non-parametric pairwise comparisons (Wilcoxon)

**Table 2:** Thickness and mechanical properties of CH and PVA-based films, alongside the average diameter of the MPs. The ImageJ software was used to measure the particle sizes of the films. The values are conveyed as mean ± standard deviation (n = 4). Significant differences (*p* > 0.05, ANOVA) are indicated by different superscript letters in the same column.

Samples	Thickness	Strain at Break	Modulus	Stress at Break	Particle Size
	mm	%	MPa	Mpa	µm
Z/CH/GM6:PVA 1:1	31.40 ± 3.13 ^b^	244.80 ± 5.70 ^f^	52.85 + 7.24 ^b^	10.15 ± 3.70 ^c^	1.05 ± 0.13 ^a,b^
Z/CH/RT6:PVA 1:2	54.60 ± 8.20 ^c,d^	151.59 ± 8.32 ^e^	12.60 ± 4.42 ^a^	4.47 ± 0.47 ^b^	1.35 ± 0.11 ^c^
Z/CH/GM6:PVA 1:3	55.75 ± 4.27 ^d^	73.27 ± 9.65 ^d^	52.82 ± 2.11 ^b^	2.68 ± 0.29 ^a^	1.13 ± 0.04 ^a,b^
Z/CH/GM6:CH 1:1	29.00 ± 5.96 ^b^	3.29 ± 1.61 ^a^	885.27 ± 92.46 ^d^	10.03 ± 2.30 ^c^	1.14 ± 0.14 ^b^
Z/CH/RT6:CH 1:2	42.33 ± 8.02 ^b,c^	19.71 ± 2.18 ^c^	735.53 ± 63.49 ^c,d^	12.34 ± 1.36 ^c^	1.50 ± 0.12 ^c^
Z/CH/RT6:CH 1:3	52.00 ± 9.27 ^c,d^	4.50 ± 2.03 ^a,b^	1827.07 ± 169.80 ^e^	12.75 ± 4.80 ^c,d^	1.57 ± 0.16 ^c^
Z/CH/GM6:CH 1:3	55.00 ± 4.00 ^d^	4.65 ± 0.86 ^a,b^	1858.80 ± 103.18 ^e^	19.02 ± 2.64 ^d^	0.92 ± 0.05 ^a^
Z/CH/GM6/CH1:2	44.33 ± 9.07 ^c,d^	22.03 ± 3.55 ^c^	648.89 ± 54.30 ^c^	16.60 ± 2.61 ^d^	1.27 ± 0.13 ^b,c^
CH	49.60 ± 3.60 ^c,d^	7.09 ± 1.52 ^b^	1879.85 ± 494.58 ^e^	22.56 ± 5.64 ^d^	______
PVA	20.25 ± 0.96 ^a^	67.43 ± 6.10 ^d^	3327.28 ± 305.92 ^f^	14.60 ± 1.47 ^c^	______

**Table 3:** Water contact angle and surface free energy for films containing varying ratios of PVA and CH, each formulated with 6% of an active compound (GM or RT). MPs with a Z:CH ratio of 5:1 are used. The mean ± standard deviation (n = 4) is used to express the values. Significant differences (*p* > 0.05, ANOVA) are indicated by different superscript letters in the same column. In this dataset, all groups shared the same superscript letter (^a^), indicating no statistically significant differences.

Sample	Water Contact Angle θ (°, t10)	SFE Total (mN/m)	SFE Disperse (mN/m)	SFE Polar (mN/m)
PVA	83.78 ± 11.81 ^a^	44.94 ± 5.25	43.10 ± 2.39	1.84 ± 2.85
CH	67.42 ± 13.49 ^a^	39.94 ± 11.49	26.18 ± 3.13	13.76 ± 8.35
Z/CH/RT6:PVA1:1	22.30 ± 6.70 ^a^	70.84 ± 7.06	36.40 ± 3.57	34.44 ± 3.49
Z/CH/RT6:PVA1:2	30.99 ± 7.38 ^a^	68.57 ± 5.33	40.33 ± 1.63	28.24 ± 3.70
Z/CH/RT6:PVA1:3	52.62 ± 13.86 ^a^	58.27 ± 14.47	43.05 ± 6.43	15.23 ± 8.04
Z/CH/GM6:PVA1:1	42.12 ± 5.31 ^a^	55.76 ± 15.30	21.96 ± 7.92	33.80 ± 7.38
Z/CH/GM6:PVA1:2	43.28 ± 28.82 ^a^	59.80 ± 22.24	35.88 ± 5.80	23.93 ± 16.44
Z/CH/GM6:PVA1:3	36.98 ± 6.42 ^a^	65.23 ± 9.76	39.78 ± 5.33	25.45 ± 4.44
Z/CH/RT6:CH1:1	54.22 ± 8.43 ^a^	55.42 ± 8.27	39.79 ± 3.28	15.62 ± 4.99
Z/CH/RT6:CH1:2	44.78 ± 9.99 ^a^	57.26 ± 8.45	32.24 ± 2.12	25.02 ± 6.33
Z/CH/RT6:CH1:3	77.80 ± 18.15 ^a^	42.05 ± 13.30	37.30 ± 6.28	4.76 ± 7.03
Z/CH/GM6:CH1:1	27.83 ± 7.17 ^a^	66.53 ± 4.75	30.85 ± 1.22	35.68 ± 3.53
Z/CH/GM6:CH1:2	67.33 ± 3.55 ^a^	48.21 ± 3.16	39.49 ± 1.39	8.72 ± 1.77
Z/CH/GM6:CH1:3	45.88 ± 6.80 ^a^	53.01 ± 11.30	36.54 ± 3.67	16.47 ± 7.63

## Data Availability

The raw data supporting the conclusions of this article will be made available by the authors on request.

## Supplementary Materials

The following supporting information can be downloaded at: https://www.mdpi.com/article/10.3390/polym18020252/s1, Figure S1: Green Mustard (GM) (a) and Red Radish var. Tango (RT) (b) extracts calibration curves; Figure S2: Comparison of UV-vis spectra of Z/CH/RT6 microparticles formulated with the RT with the active compound RT demonstrates the peak shift after encapsulation; Figure S3: Antioxidant activity of the RT and GM extracts loaded in 4 mg MPs over 168 h; Figure S4: Antioxidant activity of 4 mg loaded or unloaded MPs over 168 h; Figure S5: Films cast on Petri dishes with CH, PVA, and P as dispersing polymers and MPs as dispersed phase (MPs to DPs ratio of 1:1). The ratio of Z:CH in the MPs varied from 7:1 to 5:1; Figure S6: Optical images of films formulated using Z/CH/RT6 MPs in CH with a 1:2 ratio (a) and PVA with a 1:1 ratio (b); Figure S7: SEM images of the cryogenic fracture surfaces of CH and PVA-based formulated films; Table S1: Comparison of the present study with previous research on edible/active coatings; Table S2: Red tango (RT) and green mustard (GM) extract-encapsulated particles, with zein (Z) to chitosan (CH) ratio, and loading efficiency (LE) of the active compound; Table S3: Yield of extraction (%), total phenolic content (TPC), total flavonoid content (TFC), and % inhibition of DPPH of Green Mustard (GM) and Red Radish (RT) sprout extracts; Table S4: FTIR spectral assignments of raw materials, microparticles, and formulated films; Table S5: Thermal parameters obtained from TGA curves under nitrogen of the CH/PVA-based films.

## References

[1] Onyeaka H.N., Nwabor O.F. (2022). Chapter 9—Natural active components in smart food packaging system. Food Preservation and Safety of Natural Products.

[2] Nayanathara Thathsarani Pilapitiya P.G.C., Ratnayake A.S. (2024). The world of plastic waste: A review. Clean. Mater..

[3] Tokiwa Y., Calabia B.P., Ugwu C.U., Aiba S. (2009). Biodegradability of Plastics. Int. J. Mol. Sci..

[4] European Commission (2015). Communication from the Commission to the European Parliament, the Council, the European Economic and Social Committee and the Committee of the Regions Closing the Loop—An EU Action Plan for the Circular Economy.

[5] Karnwal A., Kumar G., Singh R., Selvaraj M., Malik T., Al Tawaha A.R.M. (2025). Natural biopolymers in edible coatings: Applications in food preservation. Food Chem. X.

[6] Moeini A., Pedram P., Fattahi E., Cerruti P., Santagata G. (2022). Edible Polymers and Secondary Bioactive Compounds for Food Packaging Applications: Antimicrobial, Mechanical, and Gas Barrier Properties. Polymers.

[7] Yadav H., Malviya R., Kaushik N. (2024). Chitosan in biomedicine: A comprehensive review of recent developments. Carbohydr. Polym. Technol. Appl..

[8] Zhang Z., Argenziano R., Konate A., Shi X., Salazar S.A., Cerruti P., Panzella L., Terrasson V., Guénin E. (2025). Preparation of chitosan/lignin nanoparticles-based nanocomposite films with high-performance and improved physicochemical properties for food packaging applications. Int. J. Biol. Macromol..

[9] Moreira B.R., Pereira-Junior M.A., Fernandes K.F., Batista K.A. (2020). An ecofriendly edible coating using cashew gum polysaccharide and polyvinyl alcohol. Food Biosci..

[10] Andrade J., González-Martínez C., Chiralt A. (2021). Effect of phenolic acids on the properties of films from Poly (vinyl alcohol) of different molecular characteristics. Food Packag. Shelf Life.

[11] Sahraee S., Milani J.M., Regenstein J.M., Kafil H.S. (2019). Protection of foods against oxidative deterioration using edible films and coatings: A review. Food Biosci..

[12] Vitiello A., Catanzano O., Guida M., Scognamiglio A., Gallo A., Costabile G., Ungaro F., Miro A., Quaglia F. (2025). Harnessing HP-β-CD in zein edible coatings for enhanced sensitive fruits preservation. Carbohydr. Polym..

[13] De Paoli M.A., Aurelio M., Waldman W. (2019). Bio-based Additives for Thermoplastics. Polímeros.

[14] Moeini A. Fungal and plant metabolites formulated into biopolymers, with anti-mold activity for food packaging. Proceedings of the International Summer School on Natural Products (ISSNP2019).

[15] Moeini A., Germann N., Malinconico M., Santagata G. (2021). Formulation of secondary compounds as additives of biopolymer-based food packaging: A review. Trends Food Sci. Technol..

[16] Aware C.B., Patil D.N., Suryawanshi S.S., Mali P.R., Rane M.R., Gurav R.G., Jadhav J.P. (2022). Natural bioactive products as promising therapeutics: A review of natural product-based drug development. S. Afr. J. Bot..

[17] Babich O., Ivanova S., Bakhtiyarova A., Kalashnikova O., Sukhikh S. (2025). Medicinal plants are the basis of natural cosmetics. Process Biochem..

[18] Masi M., Moeini S.A., Boari A., Cimmino A., Vurro M., Evidente A. (2018). Development of a rapid and sensitive HPLC method for the identification and quantification of cavoxin and cavoxone in *Phoma cava* culture filtrates. Nat. Prod. Res..

[19] Lindi A.M., Gorgani L., Mohammadi M., Hamedi S., Darzi G.N., Cerruti P., Fattahi E., Moeini A. (2024). Fenugreek seed mucilage-based active edible films for extending fresh fruit shelf life: Antimicrobial and physicochemical properties. Int. J. Biol. Macromol..

[20] Goyeneche R., Roura S., Ponce A., Vega-Gálvez A., Quispe-Fuentes I., Uribe E., Di Scala K. (2015). Chemical characterization and antioxidant capacity of red radish (*Raphanus sativus* L.) leaves and roots. J. Funct. Foods.

[21] Szőllősi R., Preedy V.R., Watson R.R. (2020). Chapter 25—Indian Mustard (*Brassica juncea* L.) Seeds in Health. Nuts and Seeds in Health and Disease Prevention.

[22] Iqbal M. (2023). Active packaging from natural ingredients applied to meat: A review. IOP Conference Series: Earth and Environmental Science.

[23] Zandi M., Ganjloo A., Bimakr M., Moradi N., Nikoomanesh N. (2021). Effect of active coating containing radish leaf extract with or without vacuum packaging on the postharvest changes of sweet lemon during cold storage. J. Food Process. Preserv..

[24] Moeini A., Salazar S.A., Gargiulo L., Kentsop R.A.D., Mattana M., Genga A., Josi C., Pedram P., Cabrera-Barjas G., Guerra S. (2026). Development of alginate-based active edible coating with *Brassica juncea* and *Raphanus sativus* sprout extracts to extend tomato shelf-life. Food Hydrocoll..

[25] Baenas N., Gómez-Jodar I., Moreno D.A., García-Viguera C., Periago P.M. (2017). Broccoli and radish sprouts are safe and rich in bioactive phytochemicals. Postharvest Biol. Technol..

[26] Mascheretti I., Alfieri M., Lauria M., Locatelli F., Consonni R., Cusano E., Dougué Kentsop R.A., Laura M., Ottolina G., Faoro F. (2021). New insight into justicidin b pathway and production in *Linum austriacum*. Int. J. Mol. Sci..

[27] Pavlátková L., Sedlaříková J., Pleva P., Peer P., Uysal-Unalan I., Janalíková M. (2023). Bioactive zein/chitosan systems loaded with essential oils for food-packaging applications. J. Sci. Food Agric..

[28] Moccia F., Agustin-Salazar S., Berg A.L., Setaro B., Micillo R., Pizzo E., Weber F., Gamez-Meza N., Schieber A., Cerruti P. (2020). Pecan (*Carya illinoinensis* (Wagenh.) K. Koch) Nut Shell as an Accessible Polyphenol Source for Active Packaging and Food Colorant Stabilization. ACS Sustain. Chem. Eng..

[29] Motalebinejad H., Bazargani-Gilani B., Pajohi-Alamoti M. (2023). Corn Zein edible film containing Sumac fruit extract and encapsulated *Thymus daenensis* Celak essential oil to improving the shelf life of chicken fillet. J. Food Meas. Charact..

[30] Mayer S., Tallawi M., De Luca I., Calarco A., Reinhardt N., Gray L.A., Drechsler K., Moeini A., Germann N. (2021). Antimicrobial and Physicochemical characterization of 2,3 Dialdehyde cellulose-based wound dressings systems. Carbohydr. Polym..

[31] Moeini A., Cimmino A., Dal Poggetto G., Di Biase M., Evidente A., Masi M., Lavermicocca P., Valerio F., Leone A., Santagata G. (2018). Effect of pH and TPP concentration on chemico-physical properties, release kinetics and antifungal activity of Chitosan-TPP-Ungeremine microbeads. Carbohydr. Polym..

[32] Mayerhöfer T.G., Pahlow S., Popp J. (2020). The Bouguer-Beer-Lambert Law: Shining Light on the Obscure. ChemPhysChem.

[33] Zhang J., Zhang J., Huang X., Arslan M., Shi J., Li Z., Gong Y., Holmes M., Zou X. (2023). Fabrication and characterization of polyvinyl alcohol/sodium alginate/zein/chitosan bilayer film for dynamic visualization of pork quality. Int. J. Biol. Macromol..

[34] Spasojević L., Katona J., Bučko S., Savić S.M., Petrović L., Budinčić J.M., Tasić C., Aidarova S., Sharipova A. (2019). Edible water barrier films prepared from aqueous dispersions of zein nanoparticles. LWT.

[35] Michiu D., Socaciu M.I., Fogarasi M., Jimborean A.M., Ranga F., Mureşan V., Semeniuc C.A. (2022). Implementation of an Analytical Method for Spectrophotometric Evaluation of Total Phenolic Content in Essential Oils. Molecules.

[36] Mania S., Banach-Kopeć A., Staszczyk K., Kulesza J., Augustin E., Tylingo R. (2023). An influence of molecular weight, deacetylation degree of chitosan xerogels on their antimicrobial activity and cytotoxicity. Comparison of chitosan materials obtained using lactic acid and CO_2_ saturation. Carbohydr. Res..

[37] Catauro M., Barrino F., Dal Poggetto G., Crescente G., Piccolella S., Pacifico S. (2020). New SiO_2_/Caffeic acid hybrid materials: Synthesis, spectroscopic characterization, and bioactivity. Materials.

[38] Abdelrazzak A.B., Hezma A.M., El-Bahy G.S. (2021). ATR-FTIR spectroscopy probing of structural alterations in the cellular membrane of abscopal liver cells. Biochim. Biophys. Acta (BBA) Biomembr..

[39] Barth A. (2007). Infrared spectroscopy of proteins. Biochim. Biophys. Acta (BBA) Bioenerg..

[40] Chiang K.Y., Matsumura F., Yu C.C., Qi D., Nagata Y., Bonn M., Meister K. (2023). True Origin of Amide I Shifts Observed in Protein Spectra Obtained with Sum Frequency Generation Spectroscopy. J. Phys. Chem. Lett..

[41] Kasaai M.R., Moosavi A. (2017). Treatment of Kraft paper with citrus wastes for food packaging applications: Water and oxygen barrier properties improvement. Food Packag. Shelf Life.

[42] Li H.M., Hu X.I.N., Guo P., Fu P., Xu L.I., Zhang X.Z. (2010). Antioxidant properties and possible mode of action of corn protein peptides and zein peptides. J. Food Biochem..

[43] Chen K., Brennan C., Cao J., Cheng G., Li L., Qin Y., Chen H. (2023). Characterization of chitosan/eugenol-loaded IRMOF-3 nanoparticles composite films with sustained antibacterial activity and their application in postharvest preservation of strawberries. LWT.

[44] Gomaa M.M., Hugenschmidt C., Dickmann M., Abdel-Hady E.E., Mohamed H.F., Abdel-Hamed M.O. (2018). Crosslinked PVA/SSA proton exchange membranes: Correlation between physiochemical properties and free volume determined by positron annihilation spectroscopy. Phys. Chem. Chem. Phys..

[45] Moeini A., Pedram P., Goudoulas T., Mehlhorn-Diehl T., Gestmann F., Fattahi E., Becker T., Germann N. (2023). Encapsulation of Neem oil from *Azadirachta indica* into Poly (lactic-co-glycolic acid) as a novel sprayable miticide system with long-term storage stability and controlled release kinetic. Ind. Crops Prod..

[46] Derkach S.R., Voron’ko N.G., Sokolan N.I., Kolotova D.S., Kuchina Y.A. (2020). Interactions between gelatin and sodium alginate: UV and FTIR studies. J. Dispers. Sci. Technol..

[47] Liu Y., Li S., Li H., Hossen M.A., Sameen D.E., Dai J., Qin W., Lee K. (2021). Synthesis and properties of core-shell thymol-loaded zein/shellac nanoparticles by coaxial electrospray as edible coatings. Mater. Des..

[48] Moeini A., Mallardo S., Cimmino A., Dal Poggetto G., Masi M., Di Biase M., van Reenen A., Lavermicocca P., Valerio F., Evidente A. (2020). Thermoplastic starch and bioactive chitosan sub-microparticle biocomposites: Antifungal and chemico-physical properties of the films. Carbohydr. Polym..

[49] Teleky B.E., Mitrea L., Plamada D., Nemes S.A., Călinoiu L.F., Pascuta M.S., Varvara R.A., Szabo K., Vodnar D.C. (2022). Development of Pectin and Poly(vinyl alcohol)-Based Active Packaging Enriched with Itaconic Acid and Apple Pomace-Derived Antioxidants. Antioxidants.

[50] Zhang X., Liu W., Liu W., Qiu X. (2020). High performance PVA/lignin nanocomposite films with excellent water vapor barrier and UV-shielding properties. Int. J. Biol. Macromol..

[51] Yun P., Devahastin S., Chiewchan N. (2021). Microstructures of encapsulates and their relations with encapsulation efficiency and controlled release of bioactive constituents: A review. Compr. Rev. Food Sci. Food Saf..

[52] Liu B., Wang Y., Yang F., Cui H., Wu D. (2018). Development of a Chlorantraniliprole Microcapsule Formulation with a High Loading Content and Controlled-Release Property. J. Agric. Food Chem..

[53] Liu G., An D., Li J., Deng S. (2023). Zein-based nanoparticles: Preparation, characterization, and pharmaceutical application. Front. Pharmacol..

[54] Zhang Y., Deng L., Guan J., Huang S., Yang J., Wu J., Li Z. Synthesis of N-alkylated Chitosan and Its Aggregation Behavior. Proceedings of the 4th Annual International Conference on Material Engineering and Application (ICMEA 2017).

[55] Salins S.S., Shetty S., Sachidananda H.K. (2025). Investigating energy absorption and crack propagation in natural rubber–epoxy composites: Design, fabrication, and fracture analysis. Polym. Bull..

[56] Singh S., Habib M., Rao E.S., Kumar Y., Bashir K., Jan S., Jan K. (2025). A comprehensive overview of biodegradable packaging films: Part I—Sources, additives, and preparation methods. Discov. Food.

[57] Gao Z., Chen G., Lu W., Wu Y., Hu B., Xu L., Fang Y., Phillips G.O. (2021). Interfacial and emulsion-stabilizing properties of zein nanoparticles: Differences among zein fractions (α-, β-, and γ-zein). Food Funct..

[58] Deng L., Kang X., Liu Y., Feng F., Zhang H. (2018). Characterization of gelatin/zein films fabricated by electrospinning vs solvent casting. Food Hydrocoll..

[59] Wang Y., Kratzer R., Murkovic M., Eibinger M., Charry E.M., Li S., Zhang T., Zhang X., Zhang M., Chen H. (2023). Fabrication and characterization of a novel zein/pectin/pumpkin seed oil Pickering emulsion and the effects of myricetin on oxidation stability. Int. J. Biol. Macromol..

[60] Matet M., Heuzey M.C., Ajji A., Sarazin P. (2015). Plasticized chitosan/polyolefin films produced by extrusion. Carbohydr. Polym..

[61] Elsherif W.M., Zayed G.M., Tolba A.O. (2024). Antimicrobial activity of chitosan- edible films containing a combination of carvacrol and rosemary nano-emulsion against Salmonella enterica serovar Typhimurium and Listeria monocytogenes for ground meat. Int. J. Food Microbiol..

