# Supplementary material for: Application of Brassica juncea and Raphanus sativus Sprout Extracts as Active Agents in Chitosan-Based Edible Coatings: Evaluation of Physicochemical and Biological Properties
Source: Polymers (Basel). 2026 Jan 16;18(2):252. doi: 10.3390/polym18020252 (PMC12845988; doi:10.3390/polym18020252)
Supplement: Supplementary file 1 [file polymers-18-00252-s001.zip › polymers-4093348-supplementary.pdf]

Supplementary Materials

Table S1. Comparison of the present study with previous research on edible/active coatings.

Study	Active Compound	Encapsulation Matrix	Film-Forming Polymer	Target Functionality	Key Advantages
**Lindi et al., 2024**	Rosemary essential oil	Not encapsulated	Fenugreek seed mucilage	Antimicrobial, antioxidant	Prolonged apple shelf life; limited structural stability
**Elsherif et al., 2024**	Carvacrol & rosemary nanoemulsion	Nanoemulsion	Chitosan	Antimicrobial	Effective for meat; no encapsulation strategy, quick release
**Iqbal, 2023**	Mustard seed extract	None	Chitosan	Antimicrobial	Applied directly; no sustained release or encapsulation
**Zandi et al., 2021**	Radish leaf extract	None	Alginate	Antioxidant	Leaf extracts used; not in sprout form or encapsulated
**This study**	*Brassica juncea* (GM) & *Raphanus sativus* (RT) sprout extracts	Zein/Chitosan microparticles	PVA and Chitosan	Antioxidant, Antimicrobial, Thermal enhancement	Novel use of sprout extracts, encapsulated for sustained release, improved thermal/mechanical properties, enhanced film homogeneity

Table S2. Red tango (RT) and green mustard (GM) extract-encapsulated particles, with zein (Z) to chitosan (CH) ratio, and loading efficiency (LE) of the active compound.

Samples names and indication	Ratio Z:CH	Loading Efficiency (%)
**Z/CH 5:1**	5:1	----
**Z/CH 5:1 RT4**	5:1	27.80%
**Z/CH 5:1 GM4**	5:1	52.09%
**Z/CH 5:1 RT6**	5:1	45.81%
**Z/CH 5:1 GM6**	5:1	41.40%
**Z/CH 5:1 RT8**	5:1	53.77%
**Z/CH 5:1 GM8**	5:1	81.25%
**Z/CH 7:1**	7:1	----
**Z/CH 7:1 RT4**	7:1	90.00%
**Z/CH 7:1 GM4**	7:1	86.30%
**Z/CH 7:1 RT6**	7:1	31.69%
**Z/CH 7:1 GM6**	7:1	50.30%
**Z/CH 7:1 RT8**	7:1	66.71%

Table S3. Yield of extraction (%), total phenolic content (TPC), total flavonoid content (TFC), and % inhibition of DPPH of Green Mustard (GM) and Red Radish (RT) sprout extracts.

Extracts	Extractive yield (%)	TPC (µg GA Eq/mg E)	TFC (µg Cat Eq/mg E)	AA (% inhibition DPPH)
RT	26.74 ± 0.66^{a}	52.0 ± 1.2^{a}	44.9 ± 2.2^{a}	16.5 ± 0.9^{a}
GM	24.05 ± 0.04^{b}	36.1 ± 0.3^{b}	41.3 ± 1.5^{a}	9.3 ± 0.5^{b}

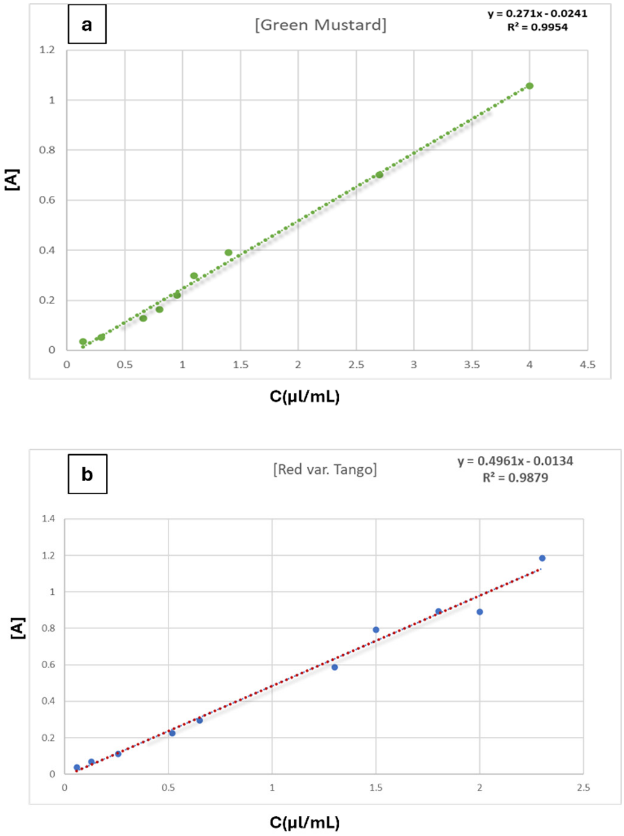

Figure S1. Green Mustard (GM) (a) and Red Radish var. Tango (RT) (b) extracts calibration curves.

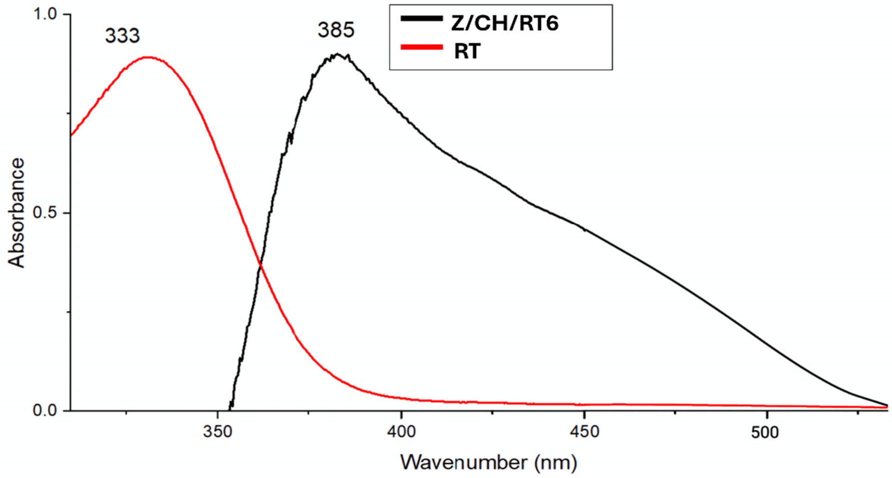

Figure S2. Comparison of UV-vis spectra of Z/CH/RT6 microparticles formulated with the RT with the active compound RT demonstrates the peak shift after encapsulation.

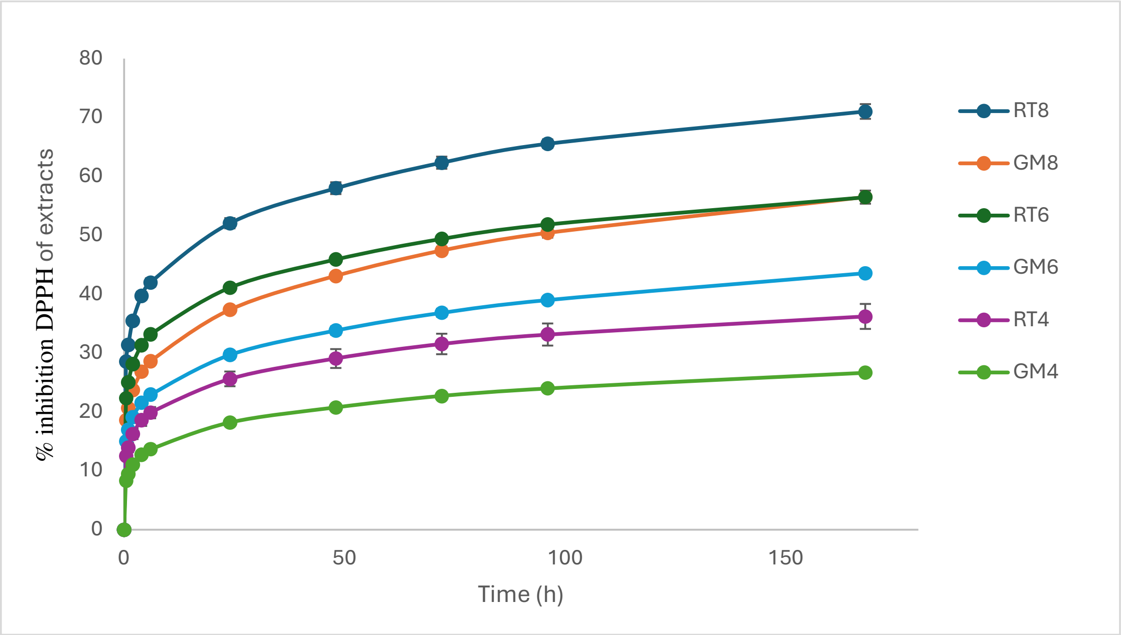

Figure S3. Antioxidant activity of the RT and GM extracts loaded in 4 mg MPs over 168 h.

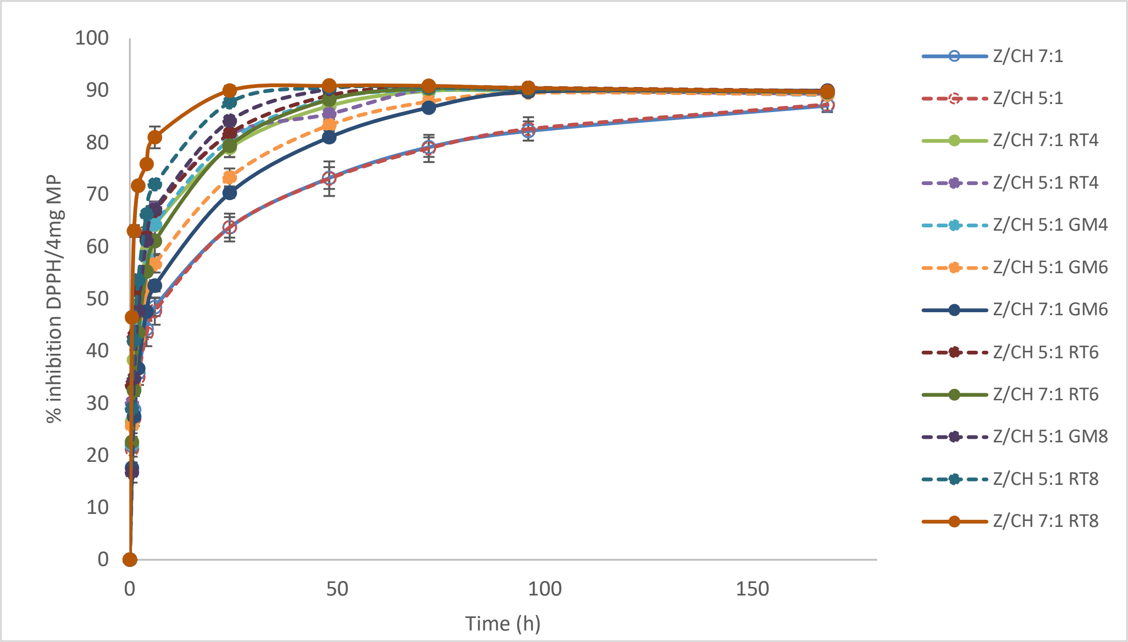

Figure S4. Antioxidant activity of 4 mg loaded or unloaded MPs over 168 h.

MPs:CH 1:1 **MPs:PVA 1:1** **MPs:Pectin 1:1**

Z:CH7:1

Z:CH5:1

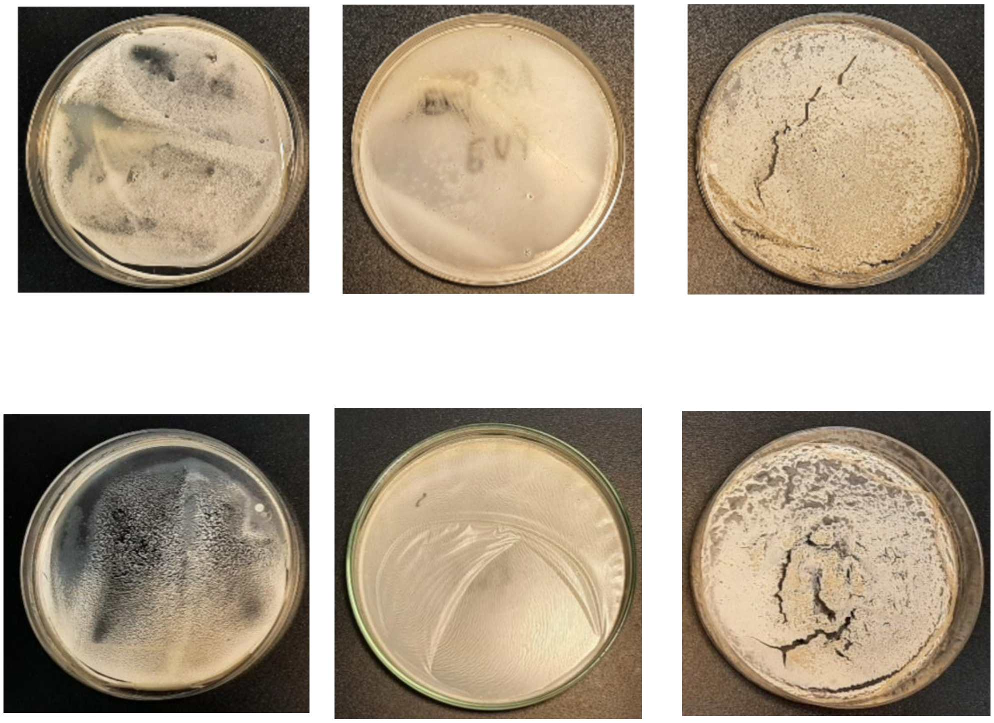

Figure S5. Films cast on Petri dishes with CH, PVA, and P as dispersing polymers and MPs as dispersed phase (MPs to DPs ratio of 1:1). The ratio of Z:CH in the MPs varied from 7:1 to 5:1.

a) Z/CH/RT6 CH 1:2 **b) Z/CH//RT6 PVA 1:1**

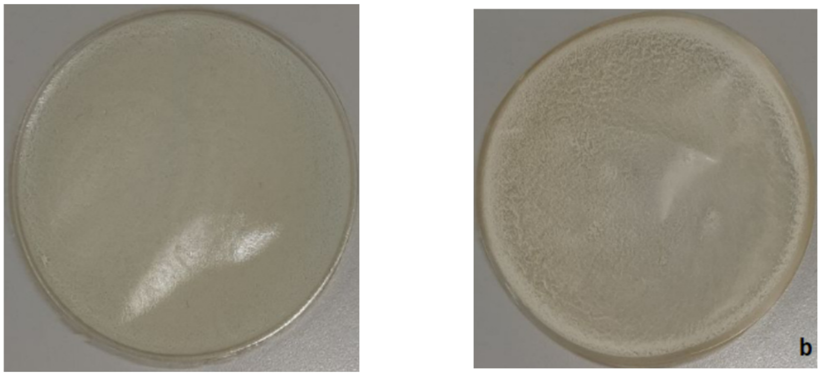

Figure S6. Optical images of films formulated using Z/CH/RT6 MPs in CH with a 1:2 ratio (a) and PVA with a 1:1 ratio (b).

Table S4. FTIR spectral assignments of raw materials, microparticles, and formulated films.

Sample	Wavenumber (cm^{-1})	Band Assignment
Zein	~3289	O–H and N–H stretching (amide A)
	~2930	C–H stretching (alkyl groups)
	~1645	C=O stretching (amide I)
	~1537	N–H bending / C–N stretching (amide II)
Chitosan	~1653	C=O stretching (amide I)
	~1534	N–H bending (amide II)
	~1070–1150	C–O–C stretching (glucosamine ring)
RT & GM extracts	~3340	O–H stretching (phenolics)
	~2923	C–H stretching (aliphatic)
	~1740	C=O stretching (esters / phenolic acids)
Z/CH MPs	~3278	H-bonded O–H/N–H stretching
	~1740	New peak: interaction between zein & Tween or RT
Formulated Films (CH/PVA + MPs)	~3300	Broad O–H stretch (enhanced H-bonding)
	~1600–1560	Amide II and phenolic interaction shift

Table S5. Thermal parameters obtained from TGA curves under nitrogen of the CH / PVA-based films.

Samples	Moisture	T_{onset}	T_{max}	Char yield
	%	°C	°C	%
**Z/CH/GM6:CH 1:1**	8.9	235.87	I) 312.76<br>II) 412.86	24.75
**Z/CH/GM6:CH 1:2**	5.08	236.1	I) 294.80<br>II) 412.50	33.25
**Z/CH/RT6:CH 1:2**	9.19	231.4	I) 296.80<br>II) 400.40	29.2
**Z/CH/GM6:CH 1:3**	12.49	211	I) 292.60<br>II) 402.40	29.54
**Z/CH/RT6:CH 1:3**	16.10	206.73	I) 291.74<br>II) 424.25	20.88
**Z/CH/GM6:PVA 1:1**	5.10	241.3	I) 321.24<br>II) 412.55	18.33
**Z/CH/RT6:PVA 1:2**	6.5	256.5	I) 316.10<br>II) 410.50	7.16
**Z/CH/GM6:PVA 1:3**	10.58	241.03	I) 320.39<br>II) 405.39	10.14
**CH**	21.05	233.12	286.82	30.92
**PVA**	9.38	263.56	I) 314.03<br>II) 437.17	9.33

Z/CH/RT6:PVA 1:2 **Z/CH/RT6:CH 1:3**

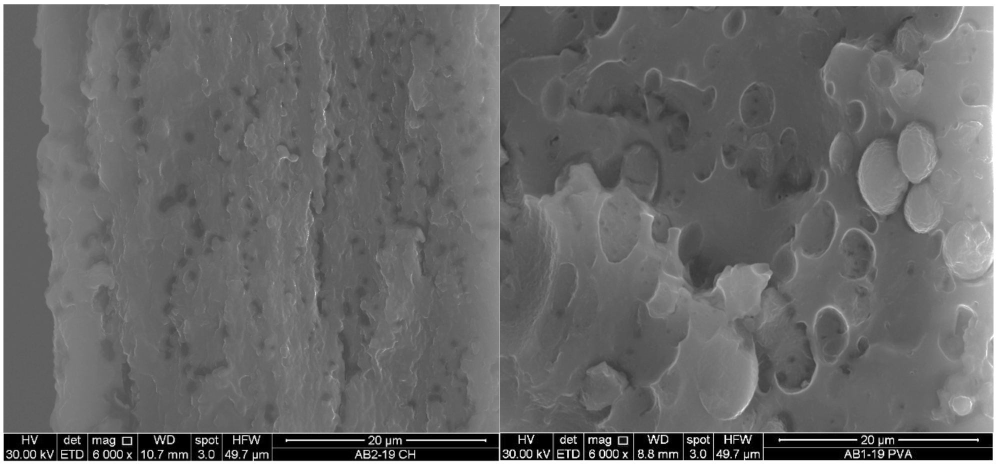

Figure S7. SEM images of the cryogenic fracture surfaces of CH and PVA-based formulated films.
